# Chronic enteritis triggered by diet westernization is driven by epithelial ATG16L1-mediated autophagy

**DOI:** 10.1080/15548627.2025.2600906

**Published:** 2026-01-05

**Authors:** Lisa Mayr, Julian Schwärzler, Laura Scheffauer, Zhigang Rao, Dietmar Rieder, Felix Grabherr, Moritz Meyer, Jakob Scheler, Almina Jukic, Luis Zundel, Verena Wieser, Andreas Zollner, Anna Simonini, Stefanie Auer, Lisa Amann, Maureen Philipp, Johannes Leierer, Richard Hilbe, Günter Weiss, Patrizia Moser, Philip Rosenstiel, Qitao Ran, Richard S. Blumberg, Arthur Kaser, Andreas Koeberle, Zlatko Trajanoski, Herbert Tilg, Timon E. Adolph

**Affiliations:** a Department of Medicine I, Gastroenterology, Hepatology, Endocrinology & Metabolism, Medical University of Innsbruck, Innsbruck, Austria; b Michael Popp Institute and Center for Molecular Biosciences Innsbruck (CMBI), University of Innsbruck, Innsbruck, Austria; c Institute of Bioinformatics, Medical University of Innsbruck, Innsbruck, Austria; d Department of Obstetrics and Gynecology, Medical University of Innsbruck, Innsbruck, Austria; e Department of Internal Medicine IV, Nephrology and Hypertension, Medical University of Innsbruck, Innsbruck, Austria; f Department of Internal Medicine II, Infectious Diseases, Immunology, Rheumatology, Pneumology, Medical University of Innsbruck, Innsbruck, Austria; g INNPATH, Innsbruck Medical University Hospital, Innsbruck, Austria; h Institute of Clinical Molecular Biology, Christian Albrecht University Kiel and Schleswig-Holstein University Hospital, Kiel, Germany; i Department of Cell Systems and Anatomy, UT Health San Antonio, San Antonio, USA; j Gastroenterology Division, Department of Medicine, Brigham and Women’s Hospital, Harvard Medical School, Boston, USA; k Division of Gastroenterology and Hepatology, Department of Medicine, Addenbrooke’s Hospital, University of Cambridge, Cambridge, UK; l Institute of Pharmaceutical Sciences and Excellence Field biohealth, University of Graz, Graz, Austria

**Keywords:** ATG16L1, Crohn disease, glutathione peroxidase 4, intestinal epithelial cells, intestinal inflammation, polyunsaturated fatty acids

## Abstract

Macroautophagy/autophagy exerts multilayered protective functions in intestinal epithelial cells (IECs) while a loss-of-function genetic variant in ATG16L1 (autophagy related 16 like 1) is associated with risk for developing Crohn disease (CD). Westernization of diet, partly characterized by excess of long-chain fatty acids, contributes to CD, and a metabolic control of intestinal inflammation is emerging. Here, we report an unexpected inflammatory function for ATG16L1-mediated autophagy in Crohn-like metabolic enteritis of mice induced by polyunsaturated fatty acid (PUFA) excess in a western diet. Dietary PUFAs induce ATG16L1-mediated conventional autophagy in IECs, which is required for PUFA-induced chemokine production and metabolic enteritis. By transcriptomic and lipidomic profiling of IECs, we demonstrate that ATG16L1 is required for PUFA-induced inflammatory stress signaling specifically mediated by TLR2 (toll-like receptor 2) and the production of arachidonic acid metabolites. Our study identifies ATG16L1-mediated autophagy in IECs as an inflammatory hub driving metabolic enteritis, which challenges the perception of protective autophagy in the context of diet westernization.

**Abbreviations**: AA: arachidonic acid; ATG16L1: autophagy related 16 like 1; CD: Crohn disease; CXCL1: C-X-C motif chemokine ligand 1; ER: endoplasmic reticulum; GFP: green fluorescent protein; GPX4: glutathione peroxidase 4; IBD: inflammatory bowel disease; IECs: intestinal epithelial cells; PTGS2/COX2: prostaglandin-endoperoxide synthase 2; PUFA: polyunsaturated fatty acid; SDA: stearidonic acid; TLR2: toll-like receptor 2; WT: wild-type

## Introduction

Crohn disease (CD) is a heterogeneous inflammatory bowel disease (IBD) in which a complex genetic underpinning and environmental cues, such as the diet, contribute to chronic unresolved intestinal inflammation [1]. For example, westernization of diet contributes to CD, a disease that demonstrated a rising incidence and prevalence across the world during industrialization over the last decades [2,3]. How the diet (and specific constituents) affect intestinal inflammation in genetically susceptible CD patients remains poorly understood [4]. For example in mice, excess of long-chain polyunsaturated fatty acids (PUFA) in a western diet instigates metabolic enteritis that is restricted by the antioxidative enzyme GPX4 (glutathione peroxidase 4) [5,6], and excess of sugar or food colorants promotes experimental colitis by perturbation of the intestinal microbiota [7,8].

Genetic variation in *ATG16L1* (autophagy related 16 like 1), and specifically the missense variant rs:41880 Thr300Ala (T300A), impairs autophagy in cell models, provides risk for developing CD [9–11], and predisposes to ileal disease [12] and a complicated disease course [13]. Autophagy deficiency in intestinal epithelial cells (IECs), induced by targeted genetic deletion of *Atg16l1*, impairs the antimicrobial effector function of specialized secretory cells in the small intestinal crypts, termed Paneth cells, and impairs regenerative responses of IECs [14,15]. Moreover, conditional deletion of *Atg16l1* in IECs evokes patchy transmural enteritis that arises consequent to endoplasmic reticulum (ER) stress and provides susceptibility to toxic colitis [16–21]. Knock-in mice that carry the human T300A variant exhibit perturbation of specialized IECs (i.e. Paneth cells and goblet cells), susceptibility to infection and IL1B (interleukin 1 beta) production [22]. As such, ATG16L1-mediated autophagy enables various protective epithelial functions but its role in intestinal epithelial metabolism during inflammation remains elusive.

As autophagy, executed by a complex machinery involving ATG16L1, is a homeostatic pathway known to control cellular metabolism beyond the intestine [23], we initially hypothesized a protective action for autophagy in IECs that counteracts stress responses elicited by diet westernization. To test this, we took advantage of a mouse model, in which excess of polyunsaturated fatty acids (PUFA) in a western diet triggers chemokine production and Crohn-like metabolic enteritis [5,6]. Surprisingly, we report that intestinal epithelial ATG16L1-mediated autophagy is required for PUFA-induced metabolic enteritis, as autophagy enables epithelial TLR2 (toll-like receptor 2)-mediated production of chemokines and metabolism of arachidonic acid-derived prostanoids.

## Results

### Dietary PUFAs induce ATG16L1-mediated autophagy in IECs

**Figure 1.**
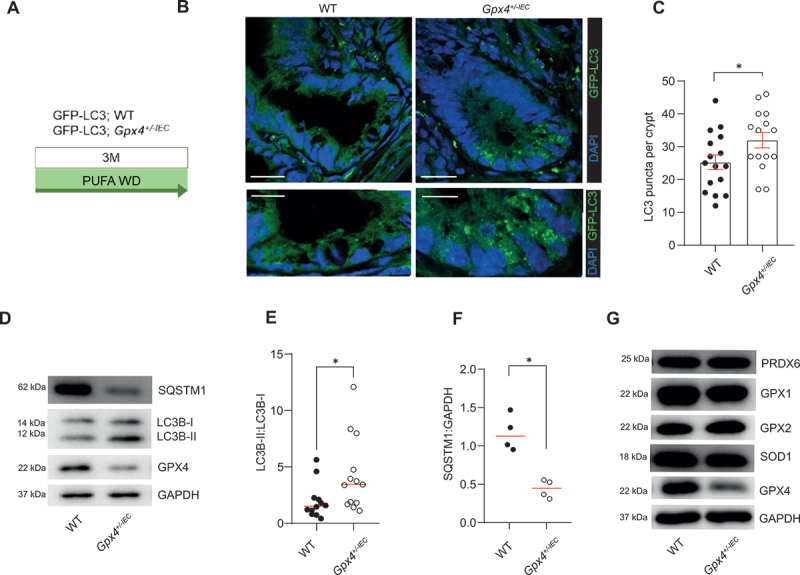
Dietary PUFAs induce autophagy in *Gpx4* deficient intestinal epithelium. (A) Experimental model of the in vivo experiment. GFP-LC3;WT and GFP-LC3;*Gpx4*^*+/-IEC*^ mice were fed a PUFA-enriched western diet for three months. (B, C) Representative confocal images (B) and quantification (C) of GFP-LC3 puncta (green) of small intestinal crypts of WT and *Gpx4*^*+/-IEC*^ mice after three months exposure to a PUFA-enriched western diet (*n* = 2). DAPI (blue) indicates nuclei. Scale bar: 10 µm (top) or 5 µm (bottom). (D-F) A representative immunoblot (D) and quantification of LC3B-II:I (E) and SQSTM1 (F) of intestinal epithelial scrapings of WT and *Gpx4*^*+/-IEC*^ mice after three months exposure to a PUFA-enriched western diet (*n* ≥4). GAPDH served as the loading control. Median is shown, Mann-Whitney test for quantification in (E, F). (G) A representative immunoblot of GPX4, SOD1, GPX2, GPX1 and PRDX6 of small intestinal epithelial scrapings of WT and *Gpx4*^*+/-IEC*^ mice after three months exposure to a PUFA-enriched western diet (*n* = 5). GAPDH served as the loading control. *p < 0.05.

**Figure 2.**
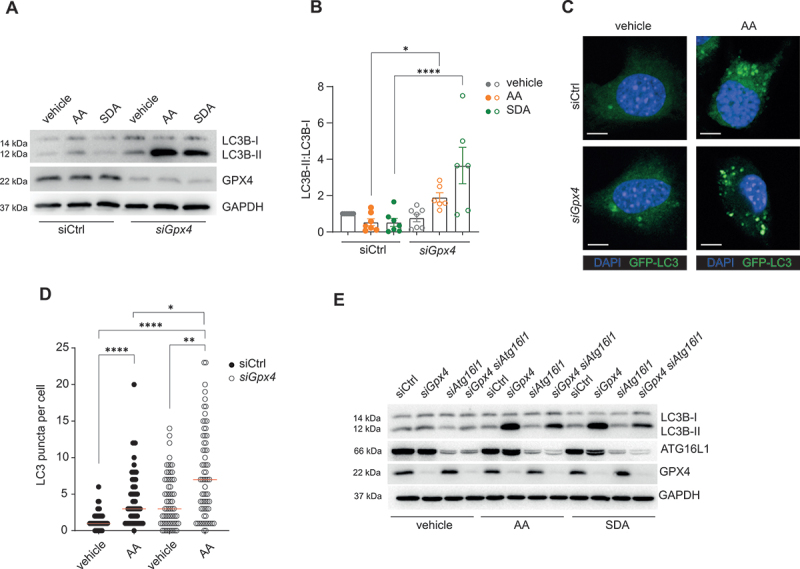
Dietary PUFAs induce autophagy in IECs restricted by GPX4. (A, B) A representative immunoblot (A) and quantification (B) of LC3B-II:I and GPX4 of siCtrl and *siGpx4* IECs after ω-6 PUFA (AA) or ω-3 PUFA (SDA) stimulation for 24 h. (*n* ≥4). GAPDH served as the loading control. (C, D) A representative confocal images (C) and quantification (D) of GFP-LC3B puncta (green) of siCtrl and *siGpx4* IECs after ω-6 PUFA (AA) stimulation for 24 h. (*n* ≥3). DAPI (blue) indicates nuclei. Scale bar: 5 µm. Median is shown, Kruskal Wallis test with Dunn’s correction for quantification in (D). (E) A representative immunoblot of LC3B-II:I, ATG16L1 and GPX4 of siCtrl, *siGpx4, siAtg16l1 and siGpx4 siAtg16l1* IECs after ω-6 PUFA (AA) or ω-3 PUFA (SDA) stimulation for 24 h. (*n* = 3). GAPDH served as the loading control. *p < 0.05, **p < 0.01, ****p < 0.0001.

By using transgenic mice that allow monitoring of autophagy, we explored whether PUFAs in a western diet induce intestinal epithelial autophagy in our model. We exposed *Gpx4*^*+/-IEC*^ mice and wild-type (WT) littermates, crossed onto GFP-LC3 reporter mice, to a PUFA-enriched western diet for 3 months (Figure 1A and Table 1) [5,6], and assessed epithelial autophagosome formation *in situ*. Indeed, a PUFA-enriched western diet induced GFP-LC3^+^ autophagosomes in IECs of *Gpx4*^*+/-IEC*^ mice as compared to WT (Figure 1B,C), which was corroborated by increased LC3B-II conversion and decreased SQSTM1/p62 in IECs from *Gpx4*^*+/-IEC*^ mice (Figure 1D–F). The expression of other antioxidative enzymes was comparable in WT and *Gpx4*^*+/-IEC*^ mice (Figure 1G and Fig. S1A), similar to the ratio of reduced glutathione (GSH) to oxidized glutathione (GSSG) in model epithelium (Fig. S1B). To define a direct role for dietary PUFAs in the induction of autophagy, we turned to the mouse small intestinal model epithelium MODE-K (“IEC”). Indeed, the ω-6 PUFA arachidonic acid (AA) and the ω-3 PUFA stearidonic acid (SDA), which are contained in the PUFA-enriched western diet, induced autophagy in IECs with reduced *Gpx4* expression (mediated by siRNA, Fig. S1C-F) [5,6], as indicated by a GFP-LC3 reporter assay and by LC3B-II conversion (Figure 2A–D). Moreover, PUFA-induced autophagy in *Gpx4*-deficient IECs required *Atg16l1* (Figure 2E and Fig.. S1G,H). Collectively, these data indicate that dietary PUFAs induce ATG16L1-mediated autophagy in *Gpx4*-deficient IECs.

**Table 1.** Fatty acid composition in PUFA-enriched wd (ssniff, TD88137 + 10% fish oil).

Fatty acid composition	% of diet
C 4:0	0.57
C 6:0	0.38
C 8:0	0.20
C 10:0	0.45
C 12:0	0.54
C 14:0	2.33
C 16:0	5.84
C 17:0	0.15
C 18:0	1.85
C 20:0	0.08
C 16:1	1.11
C 18:1	4.45
C18:2 (ω-6)	0.41
C18:3 (ω-3)	0.15
C 18:4 (ω-3)	0.3
C 20:4 (ω-6)	0.09
C 20:5 (ω-3)	1.75
C22:5 (ω-3)	0.19
C22:6 (ω-3)	1.21

### Epithelial ATG16L1-mediated autophagy enables PUFA-induced enteritis

**Figure 3.**
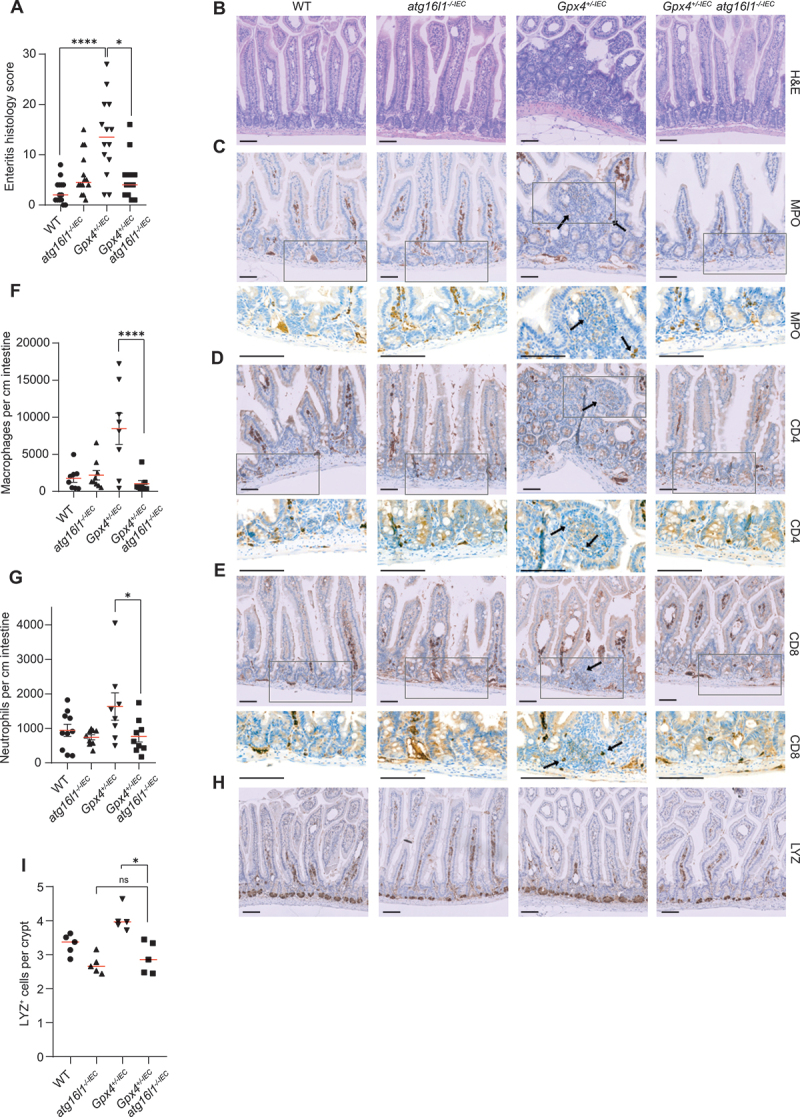
Epithelial-specific deletion of *Atg16l1* in mice protected against metabolic enteritis induced by dietary PUFAs. (A, B) Enteritis histology score (A) and representative H&E images (B) of WT, *Gpx4*^*+/-IEC*^, *atg16l1*^*-/-IEC*^ and *Gpx4*^*+/-IEC*^;*atg16l1*^*-/-IEC*^ mice after three months exposure to a PUFA-enriched western diet (*n* = 14/14/14/13). Median is shown, each dot represents an experimental animal, Kruskal-Wallis test with Dunn’s correction (A). Scale bar: 100 µm in (B). (C-E) Representative images of MPO^+^ neutrophils (C), CD4^+^ (D) and CD8^+^ T cells (E) of intestinal sections from WT, *Gpx4*^*+/-IEC*^, *atg16l1*^*-/-IEC*^ and *Gpx4*^*+/-IEC*^;*atg16l1*^*-/-IEC*^ mice after three months exposure to a PUFA-enriched western diet. Scale bar: 100 µm. Black arrows indicate MPO positive cells (C), CD4 positive cells (D) and CD8 positive cells (E) in patchy inflammatory spots of *Gpx4*^*+/-IEC*^ mice. (F, G) Quantification of macrophages (F) and neutrophils (G) per cm small intestine of WT, *Gpx4*^*+/-IEC*^, *atg16l1*^*-/-IEC*^ and *Gpx4*^*+/-IEC*^;*atg16l1*^*-/-IEC*^ mice after three months exposure to a PUFA-enriched western diet (*n* ≥8). (H, I) Representative immunohistochemistry (H) and quantification (I) of LYZ^+^ (lysozyme^+^) cells in the small intestine of WT, *Gpx4*^*+/-IEC*^, *atg16l1*^*-/-IEC*^ and *Gpx4*^*+/-IEC*^;*atg16l1*^*-/-IEC*^ mice after three months exposure to a PUFA-enriched western diet (*n* = 5). Scale bar: 100 μm (H). Median is shown, each dot represents an experimental animal. Kruskal-Wallis test with Dunn’s correction (I). *p < 0.05, ****p < 0.0001.

**Figure 4.**
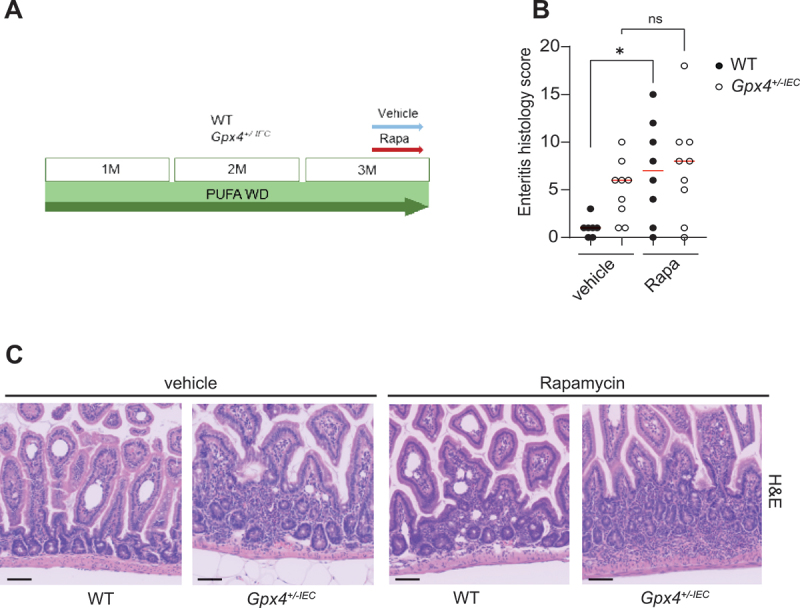
Rapamycin enables PUFA-induced enteritis in WT mice. (A) Experimental model of the in vivo experiment. WT and *Gpx4*^*+/-IEC*^ were fed a PUFA-enriched western diet for three months and treated with vehicle or rapamycin (Rapa) for the last two weeks of the experiment. (B, C) enteritis histology score (B) and representative H&E images (C) of WT and *Gpx4*^*+/-IEC*^ fed a PUFA WD for three months and treated with vehicle or rapamycin (Rapa) for the last two weeks of the experiment (*n* = 7/9/8/9). Median shown, each dot represents an experimental animal. Kruskal-Wallis test with Dunn’s correction (B). Scale bar: 100 µm (C). *p < 0.05.

Next, we explored whether PUFA-induced autophagy controls enteritis in mice. Feeding a PUFA-enriched western diet for 3 months elicited patchy discontinuous chronic enteritis with infiltration of MPO^+^ neutrophils and CD4^+^ and CD8^+^ T cells in *Gpx4*^*+/-IEC*^ mice as compared to controls (Figure 3A–G and Fig. S1I). We generated *Gpx4*^*+/-IEC*^ mice that co-deleted both alleles of *Atg16l1* in IECs (*Gpx4*^*fl/WT*^;*atg16l1*^*fl/fl*^;*Vil1Cre*^*+*^ mice), leading to intestinal epithelial autophagy deficiency (Fig. S1J,K) [18], and compared disease severity of these double mutant mice to that of *Gpx4*^*+/-IEC*^ mice after exposure to a PUFA-enriched western diet. Notably, *Gpx4*^*+/-IEC*^;*atg16l1*^*-/-IEC*^ double mutant mice were protected against PUFA-induced enteritis and against systemic inflammation when compared to *Gpx4*^*+/-IEC*^ mice (Figure 3A–G and Fig. S1L). By contrast, there was no difference in weight or blood glucose concentration between the genotypes (Fig S1M,N). Paneth cell defects, indicated by impaired lysozyme expression, were comparable between *Gpx4*^*+/-IEC*^;*atg16l1*^*-/-IEC*^ and *atg16l1*^*-/-IEC*^ mice (Figure 3H,I). In turn, we tested whether induction of intestinal epithelial autophagy with intraperitoneal injection of rapamycin (which also exerts potent immunosuppressive functions [18,24]) affected susceptibility to enteritis in our model. Notably, rapamycin treatment failed to rescue enteritis in *Gpx4*^*+/-IEC*^ mice, but induced enteritis in WT mice exposed to a PUFA-enriched western diet (Figure 4A–C). These studies demonstrate that intestinal epithelial ATG16L1-mediated autophagy exerts inflammatory actions in the gut during exposure to a PUFA-enriched western diet.

### PUFA-induced autophagy is required for chemokine production in IECs

**Figure 5.**
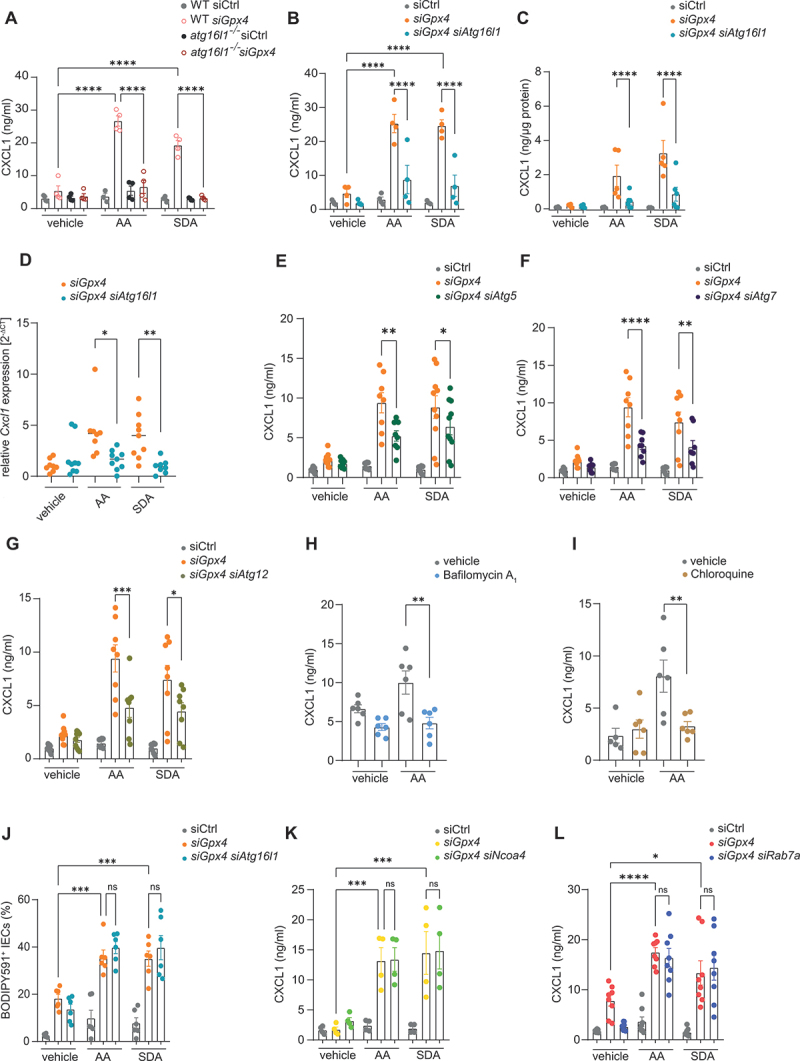
ATG16L1-mediated autophagy is required for PUFA-induced CXCL1 production in IECs. (A) Quantification of CXCL1 in the supernatant of siCtrl and *siGpx4* of WT and *atg16l1*^*-/-*^ IECs after ω-6 PUFA (AA) or ω-3 PUFA (SDA) stimulation for 24 h. (*n* = 4). (B – D) Quantification of CXCL1 in the supernatant (B), protein lysate (C) and relative *Cxcl1* expression (D) of siCtrl, *siGpx4* and *siGpx4 siAtg16l1* IECs after ω-6 PUFA (AA) or ω-3 PUFA (SDA) stimulation for 24 h. (*n* = 4/6). (E – G) Quantification of CXCL1 in the supernatant of siCtrl, *siGpx4* and *siGpx4 siAtg5* (E), *siGpx4 siAtg7* (F) and *siGpx4 siAtg12* (G) IECs after ω-6 PUFA (AA) or ω-3 PUFA (SDA) stimulation for 24 h (*n* > 3). Note that vehicle and siCtrl and *siGpx4* controls are identical in (E – G) as experiments were performed at the same time. (H, I): Quantification of CXCL1 in the supernatant of *siGpx4* IECs after ω-6 PUFA (AA) or ω-3 PUFA (SDA) stimulation for 24 h with or without bafilomycin A_1_ (H) or chloroquine (I) (*n* = 3). (J) Quantification of lipid peroxidation by flow cytometry of BODIPY 581/591 C11^+^ labelled siCtrl, *siGpx4* and *siGpx4 siAtg16l1* IECs after ω-6 PUFA (AA) or ω-3 PUFA (SDA) stimulation for 24 h (*n* = 3). (K, L) quantification of CXCL1 in the supernatant of siCtrl, *siGpx4* and *siGpx4 siNcoa4* (K) and siCtrl, *siGpx4* and *siGpx4 siRab7a* (L) after ω-6 PUFA (AA) or ω-3 PUFA (SDA) stimulation for 24 h (*n* ≥4). *p < 0.05, **p < 0.01, ***p < 0.001, ****p < 0.0001.

Next, we tested whether autophagy was required for PUFA-induced chemokine production in our model. We generated *Atg16l1*-deficient IECs by CRISPR-Cas9 gene editing, which indeed abrogated rapamycin-induced autophagy (Fig. S2A-C). We assessed the impact of *Atg16l1*-deficiency on PUFA-induced CXCL1 (C-X-C motif chemokine ligand 1) expression, as this chemokine (besides CXCL2) was potently induced in our model system and contributes to enteritis in *Gpx4*^*+/-IEC*^ mice [6]. Importantly, *Atg16l1*-deficiency blunted CXCL1 production elicited by PUFA exposure in *siGpx4* IECs (Figure 5A). Likewise, PUFA-induced CXCL1 production was abrogated by transcriptional means in *siGpx4* IECs in which we co-silenced *Atg16l1* with siRNA (Figure 5B–D). Importantly, co-silencing of *Atg5*, *Atg7* or *Atg12* and other core autophagy genes, such as *Becn1* and *Rb1cc1/Fip200*, protected against PUFA-induced CXCL1 production in *siGpx4* IECs, indicating that conventional autophagy controls the inflammatory tone in our model (Figure 5E–G and Fig. S2D-H). In line with this, treatment with bafilomycin A_1_ or chloroquine (blocking autophagic flux [25]) blunted PUFA-induced CXCL1 production in *siGpx4* IECs (Figure 5H,I). By contrast, *Atg16l1*-deficiency did not affect PUFA-induced lipid peroxidation in *siGpx4* IECs (Figure 5J), and co-silencing of receptors for ferritinophagy (*Ncoa4*) or lipophagy (*Rab7a, Vps4a and Spart/Spg20*) did not affect PUFA-induced CXCL1 production (Figure 5K,L and Fig. S3A-C).

Finally, we also generated IECs carrying the human T300A *ATG16L1* variant (Fig. S4A-D) and confirmed the validity of this model by increased susceptibility to CASP3 (caspase 3)-mediated cleavage of mutant ATG16L1 (Fig. S4E,F) [9]. The T300A mutation resides in the WD domain of ATG16L1 and impairs selective (but not conventional) autophagy [26], while providing risk for CD [10]. IECs carrying the T300A *ATG16L1* variant did not affect PUFA-induced CXCL1 production in our model, as compared to IECs carrying the human wild-type variant (Fig. S4G). Collectively, these data indicated that the process of conventional autophagy is required for PUFA-induced CXCL1 production in this model.

### Intestinal epithelial ATG16L1 is required for TLR2 activity driving PUFA-induced enteritis

**Figure 6.**
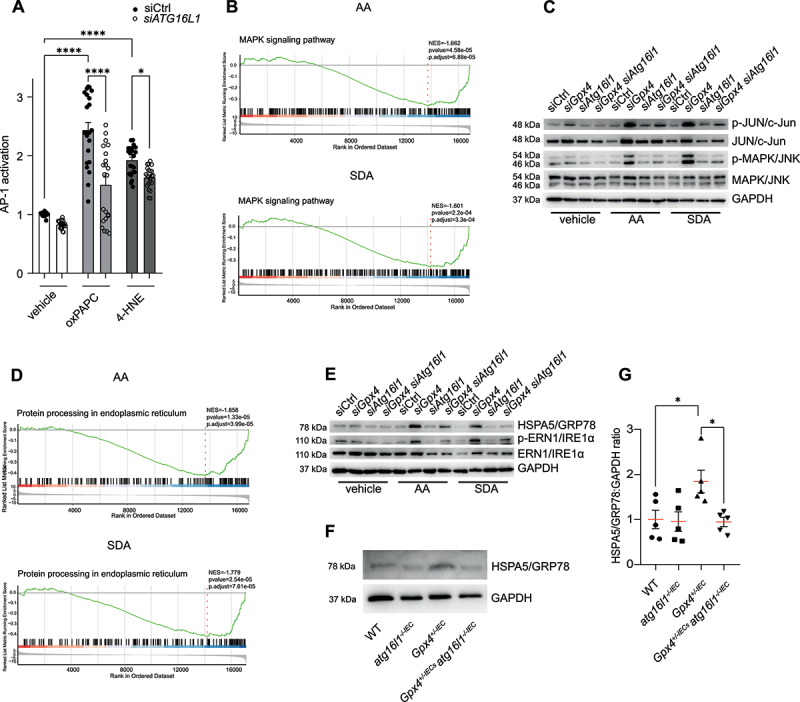
*Atg16l1* is required for PUFA-induced TLR2 activity in IECs. (A) Relative AP-1 activation in siCtrl or *siATG16L1* mTLR2 reporter cell line after stimulation with vehicle, oxidized phosphatidylcholine (oxPAPC) or the lipid peroxidation by-product 4-hydroxynonenal (4-HNE) for 24 h (*n* = 8). (B) Enrichment plots showing significant downregulation of MAPK signaling pathway in AA and SDA stimulated IECs. *siGpx4 siAtg16l1* IECs were compared to *siGpx4* IECs (*n* = 4). Red and blue color bar: this bar corresponds to the ranked list of genes. * red: indicates genes in the MAPK signaling pathway that are upregulated in *siGpx4 siAtg16l1* +AA/+SDA compared to *siGpx4* +AA/+SDA. Blue: indicates genes in the MAPK signaling pathway that are downregulated in *siGpx4 siAtg16l1* +AA/+SDA compared to *siGpx4* +AA/+SDA. The intensity of the color reflects the degree of upregulation or downregulation. (C) A representative immunoblot of (phospho-)MAPK/JNK and (phospho-) JUN/c-Jun in siCtrl, *siGpx4*, *siAtg16l1* and *siGpx4 siAtg16l1* IECs after ω-6 PUFA (AA) or ω-3 PUFA (SDA) stimulation for 24 h. (*n* ≥4). GAPDH served as the loading control. (D) Enrichment plots showing significant downregulation of the protein processing at the endoplasmic reticulum pathway in AA and SDA stimulated IECs. *siGpx4 siAtg16l1* IECs were compared to *siGpx4* IECs (*n* = 4). Red and blue color bar: this bar corresponds to the ranked list of genes. * red: indicates genes in the protein processing at the endoplasmic reticulum pathway that are upregulated in *siGpx4 siAtg16l1* +AA/+SDA compared to *siGpx4* +AA/+SDA. Blue: indicates genes in the protein processing at the endoplasmic reticulum pathway that are downregulated in *siGpx4 siAtg16l1* +AA/+SDA compared to *siGpx4* +AA/+SDA. The intensity of the color reflects the degree of upregulation or downregulation. (E) a representative immunoblot of (phospho-)ERN1/IRE1α and HSPA5/GRP78 in siCtrl, *siGpx4*, *siAtg16l1* and *siGpx4 siAtg16l1* IECs after ω-6 PUFA (AA) or ω-3 PUFA (SDA) stimulation for 24 h. (*n* ≥4). GAPDH served as the loading control. (F, G) A representative immunoblot (F) and quantification by densitometry (G) of epithelial HSPA5/GRP78 relative to GAPDH of WT, *Gpx4*^*±IEC*^, *atg16l1*^*-/-IEC*^ and *Gpx4*^*±IEC*^;*atg16l1*^*-/-IEC*^ mice after three months exposure to a PUFA-enriched western diet (*n* = 5). Each dot represents one experimental animal. GAPDH served as the loading control in F. *p < 0.05, ****p < 0.0001.

We further studied how *Atg16l1* governed PUFA-induced chemokine production in *Gpx4*-deficient IECs and whether such a mechanism is relevant for PUFA-induced enteritis. Bulk RNA sequencing of PUFA-stimulated IECs indicated transcriptional evidence for ER stress and mitogen-activated protein kinase (MAPK) activity in *siGpx4* IECs, which was abrogated in *Atg16l1*-deficient *siGpx4* IECs (Fig. S5A,B and Fig. S6A,B), similar to other cellular pathways (Fig. S6C,D). As we previously demonstrated that PUFA-induced lipid peroxidation mediated TLR2 activity and consequently ER stress and MAPK signaling in *Gpx4*-deficient IECs [6], we hypothesized that ATG16L1 was required for PUFA-induced TLR2 activity in our model. Indeed, a reporter cell line indicated that ATG16L1 was required for TLR2 activity induced by lipid peroxidation by-products (Figure 6A). In line, we noted reduced evidence of TLR2-mediated (i.e. stress-related) MAPK signaling (Figure 6B), indicated by reduced MAPK/JNK phosphorylation, in *Atg16l1*-deficient *siGpx4* IECs after PUFA exposure, when compared to *siGpx4* IECs (Figure 6C and Fig. S6E-H). Likewise, *Atg16l1*-deficient *siGpx4* IECs exhibited reduced signs of ER stress (downstream of TLR2) after PUFA exposure (Figure 6D), indicated by reduced ERN1/IRE1 phosphorylation and HSPA5/GRP78 expression (Figure 6E–G and Fig. S6I,J). These observations indicated that ATG16L1 was required for PUFA-induced TLR2 activity and related inflammatory stress signaling in our model.

**Figure 7.**
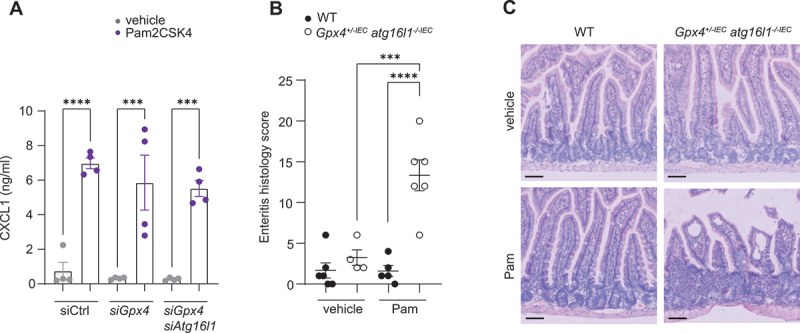
Pam2CSK4 induces inflammation in *Gpx4*^*+/-IEC*^; *atg16l1*^*-/-IECs*^ mice. (A) Quantification of CXCL1 in the supernatant of siCtrl, *siGpx4* and *siGpx4 siAtg16l1* IECs with or without stimulation with the TLR2 agonist Pam2CSK4 for 24 h (*n* = 4). (B, C) Enteritis histology score (B) and representative H&E images (C) of WT and *Gpx4*^*+/-IEC*^;* atg16l1*^*-/-IECs*^ fed a PUFA-enriched western diet for three months and treated with vehicle or Pam2CSK4 (Pam) for the last week of the experiment (*n* = 6/4/5/6). Scale bar: 100 µm in C. ***p < 0.001, ****p < 0.0001.

As we previously demonstrated that genetic deletion of *Tlr2* and pharmacological blockade of MAPK/JNK signaling contributed to PUFA-induced CXCL1 production and enteritis in this model [6], we tested whether stimulation with the TLR2 agonist Pam2CSK4 was able to induce TLR2 activity in *Atg16l1*-deficient IECs. Indeed, Pam2CSK4 induced CXCL1 production in *Atg16l1*-deficient *siGpx4* IECs (Figure 7A). Moreover, Pam2CSK4 gavage into *Gpx4*^*+/-IEC*^;*atg16l1*^*-/-IEC*^ mice (for 7 consecutive days in the last week of exposure to a PUFA-enriched western diet for 3 months) was sufficient to instigate enteritis in this model (Figure 7B,C). These studies indicated that induction of TLR2 activity with a prototypical TLR2 ligand (Pam2CSK4) evoked CXCL1 production in *Atg16l1*-deficient IECs and enteritis in our model, suggesting that ATG16L1 controlled TLR2 activity upstream of this receptor while being dispensable for execution of TLR2 (downstream) signaling.

### ATG16L1 is required for PTGS2/COX2-mediated arachidonic acid metabolism in IECs

**Figure 8.**
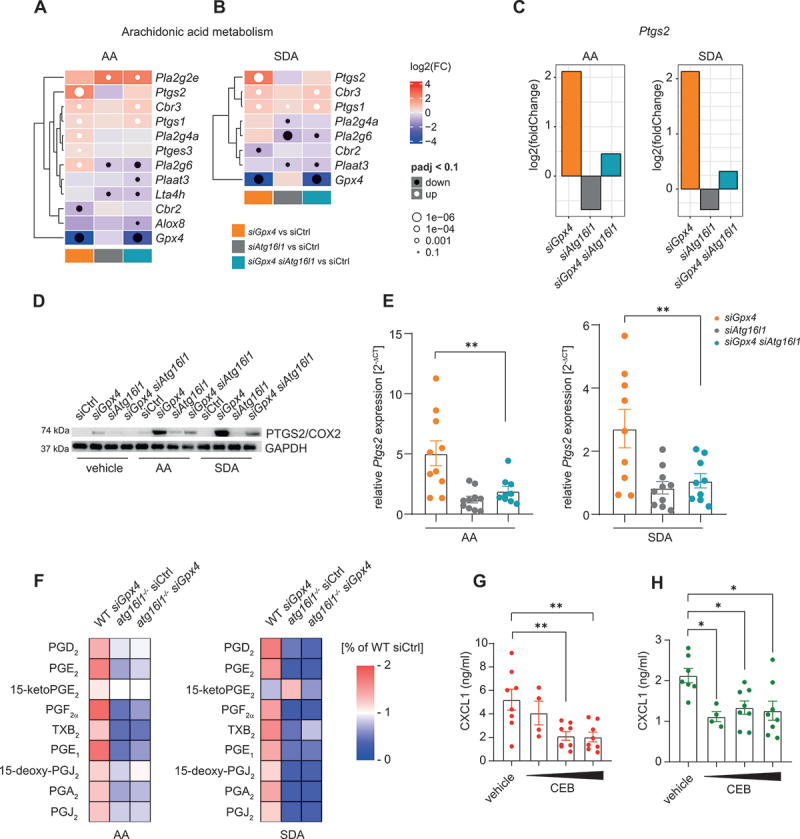
*Atg16l1* is required for PTGS2/COX2-mediated AA metabolism in IECs. (A, B) Heatmap showing significant altered expression of AA metabolism genes in *siGpx4*, *siAtg16l1* and *siGpx4 siAtg16l1* IECs after stimulation with ω-6 PUFA (AA, A) or ω-3 PUFA (SDA, B) for 8 h compared to siCtrl. AA metabolism genes are indicated on the right. (C) *Ptgs2* Fold change in *siGpx4*, *siAtg16l1* and *siGpx4 siAtg16l1* IECs after exposure with ω-6 PUFA (AA) or ω-3 PUFA (SDA) for 8 h compared to siCtrl. (D) A representative immunoblot of PTGS2/COX2 in siCtrl, *siGpx4*, *siAtg16l1* and *siGpx4 siAtg16l1* IECs after ω-6 PUFA (AA) or ω-3 PUFA (SDA) exposure for 24 h. (*n* = 3). GAPDH served as the loading control. (E) Relative expression of *Ptgs2* relative to *Actb/β-actin* in *siGpx4*, *siAtg16l1* and *siGpx4 siAtg16l1* IECs after ω-6 PUFA (AA) or ω-3 PUFA (SDA) stimulation for 8 h determined by qPCR (*n* ≥4). (F) Levels of lipid mediators in vehicle, AA or SDA exposed *atg16l1*^*-/-*^ IECs relative to WT IECs. The heatmap shows the percentage changes of *atg16l1*^*-/-*^ IECs and wt IECs compared to wt siCtrl IECs (*n* = 4). (g, H) Quantification of CXCL1 in the supernatant of *siGpx4* IECs after ω-6 PUFA (AA, G) or ω-3 PUFA (SDA, H) stimulation for 24 h with the PTGS2/COX2 inhibitor celecoxib (0.1 µM, 1 µM, 10 µM, CEB) (*n* = 4). *p < 0.05, **p < 0.01.

Finally, we studied whether ATG16L1-mediated autophagy controlled PUFA metabolism in our model, because (I) oxidative arachidonic acid metabolism is known to control immune cell activation and function, including inflammatory responses of macrophages [27], and (II) PUFAs or related metabolites could be involved in the control of TLR2 activity, and thus the expression of downstream target genes, for example *Ptgs2/Cox2* (prostaglandin-endoperoxidase synthase 2) [28,29]. Bulk RNA sequencing after AA or SDA stimulation indicated increased expression of *Ptgs2* in *siGpx4* IECs, when compared to siCtrl IECs, which was abrogated in *Atg16l1*-deficient *siGpx4* IECs (Figure 8A–C). By contrast, most other PUFA-metabolizing enzymes for example PTGS1/COX1, were not differentially expressed in *siGpx4* IECs when compared to *Atg16l1*-deficient *siGpx4* IECs (Fig. S7A and B). Next, we confirmed that *Atg16l1*-deficient *siGpx4* IECs exhibited markedly reduced PTGS2/COX2 expression when compared to *siGpx4* IECs after PUFA exposure by Western blot (Figure 8D and Figure S7C-E), and by quantitative PCR (Figure 8E), but not *siAtg16l1* IECs (Fig. S7D,E). These studies led us to hypothesize that ATG16L1 controls PUFA metabolism and specifically PTGS2/COX2-mediated arachidonic acid metabolism in IECs.

We addressed this hypothesis by analyzing the PUFA-derived lipid mediator profile of IECs exposed to AA (20:4), SDA (18:4) or vehicle for 30 min using ultra high-performance liquid chromatography-tandem mass spectrometry (UPLC-MS/MS). The supplementation of AA increased the absolute amount and relative proportion of 20:4 and its elongation product docosatetraenoic acid (22:4) in phosphatidylcholine (PC) and phosphatidylethanolamine (PE) species in *siGpx4* IECs with or without *Atg16l1*-deficiency (Fig. S7F). Accordingly, the supplementation of SDA increased the abundance of the elongation/desaturation product eicosapentaenoic acid (20:5) in phospholipids, indicating the validity of our approach. More importantly, *Atg16l1*-deficient *siGpx4* IECs were suppressed in their ability to generate COX products from arachidonic acid, i.e., the prostanoids PGE_2_, PGD_2_, PGF_2α_, PGA_2_, PGJ_2_ and TXB_2_ and the monohydroxylated fatty acid 11-HETE, when compared to *siGpx4* IECs (Figure 8F and Fig. S8A-I), which was associated with an increased abundance of AA in the supernatant (Fig. S9A). No significant changes were observed in other metabolites such as LTB_4_, 5-HETE or 15-HETE (among others) (Fig. S9B,C). These studies demonstrated that ATG16L1 markedly influences PUFA metabolism in IECs and specifically the generation of AA-derived prostanoids, likely by controlling PTGS2/COX2 expression. The PTGS2/COX2 inhibitor celecoxib (CEB) reduced PUFA-induced CXCL1 production in si*Gpx4* IECs (Figure 8G,H), suggesting a link between prostanoid biosynthesis and proinflammatory chemokine production in our model.

## Discussion

Environmental cues elicited by westernization of diet are contributing to CD, while specific examples and mechanisms for such a concept remain scarce [3,30]. For example, we previously demonstrated that CD epithelium responds to dietary PUFA excess with an inflammatory response, which is determined by GPX4, and we demonstrated that PUFA excess correlates with a poor course of CD [6]. Despite these (pre)clinical observations, a specific molecular machinery controlling PUFA metabolism and related metabolic inflammation in intestinal epithelium remains elusive. Here, we determined that dietary PUFAs induce ATG16L1-mediated intestinal epithelial autophagy, which is required for PUFA-induced chemokine production and metabolic enteritis in a mouse model that recapitulates aspects of human CD. Bulk RNA sequencing of PUFA-exposed model IECs suggested that ATG16L1 is required for TLR2-mediated stress signaling and enteritis, which we corroborated in reporter cells and mice. Moreover, RNA sequencing and lipidomic profiling of PUFA-exposed IECs indicate that ATG16L1 is required for PTGS2/COX2 expression and prostanoid formation, which likely exert inflammatory actions [31], and PTGS2/COX2-related lipid mediators are thought to contribute to intestinal inflammation in IBD [32]. As such, we report an unexpected inflammatory action for ATG16L1-mediated autophagy in the context of PUFA excess challenging susceptible epithelium in the intestine, without affecting systemic metabolism (indicated by body weight and blood glucose concentration) [33]. This action is unexpected because several studies demonstrated the protective actions of autophagy in epithelium specifically in the intestine (without PUFA exposure) (e.g [16–19,34,35].), and because autophagy has been implicated in the control of metabolic inflammation beyond the gut, in part by removal of inflammatory cellular content [36]. More specifically, the same mouse model (i.e., *atg16l1*^*-/-IEC*^ mice) is susceptible to colitis and enteritis (induced by dextran sodium sulfate colitis or by genetic deletion of *Xbp1* (X-box binding protein 1) [18], while protecting against PUFA-induced metabolic enteritis in *Gpx4*^*+/-IEC*^ mice, as reported herein. As such, ATG16L1-mediated intestinal epithelial autophagy exerts protective or deleterious actions that are context specific and can be related to diet westernization. Beyond the intestine, pro-inflammatory actions of autophagy have been reported, for example during infection [37] and sepsis [38].

GPX4 protects against a regulated form of iron-dependent cell death termed ferroptosis that is controlled by redox homeostasis of PUFAs contained in PE and PC (in cellular membranes), and that relies on autophagy [39]. Although epithelial cell death is not phenotypically characterizing enteritis in intestinal-epithelial-specific *Gpx4*-deficient mice with or without PUFA exposure [5,6], we found that ATG16L1-mediated autophagy is required for the inflammatory phenotype, as similarly reported for ferroptosis [40]. More specifically, autophagy was required for ferroptosis of various cell models [41], as exemplified by lipophagy (*Rab7a*-mediated autophagy of lipid droplets) that is required for RSL3-induced ferroptosis in hepatocytes [42] or ferritinophagy (*Ncoa4*-mediated autophagy of ferritin) [43]. How autophagy is involved in the execution of ferroptosis is currently debated but could involve the control of lipid peroxidation [44]. In our model of intestinal epithelial *Gpx4*-deficiency, PUFA-induced ATG16L1-mediated conventional autophagy did not affect lipid peroxidation and *Rab7a* (and other lipophagy receptors) and *Ncoa4* were dispensable for CXCL1 production. We rather report a mechanism by which *Atg16l1*-deficiency blunted PUFA-induced TLR2 activity and related stress signaling in *Gpx4*-deficient IECs. By contrast, the prototypical TLR2 ligand Pam2CSK4 was still able to induce an inflammatory response from *Atg16l1*-deficient IECs, and Pam2CSK4 instigated enteritis in our model. These findings suggest that ATG16L1-mediated autophagy is required for the generation of an inflammatory metabolite in IECs upon PUFA exposure, while autophagy appears dispensable for the execution of classical TLR2 signaling.

**Figure 9.**
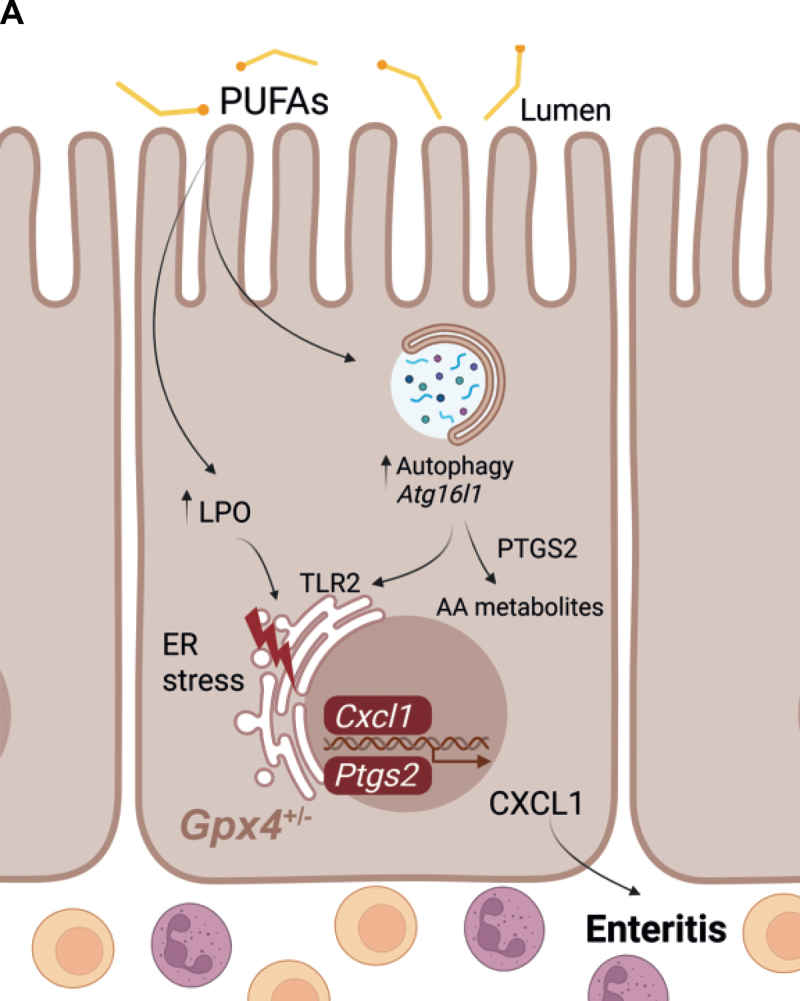
Model depicting PUFA-induced actions in *Gpx4*-deficient intestinal epithelium. PUFAs induce ATG16L1-mediated conventional autophagy which enables metabolic enteritis originating from *Gpx4*-deficient IECs. Specifically, ATG16L1 enables TLR2 activation and arachidonic acid metabolism into prostanoids. Image was created with BioRender.com.

A limitation of our study is that we cannot resolve how autophagy specifically controls TLR2 activity, although recent evidence suggests that PUFA metabolites control the inflammatory tone and possibly toll-like receptor activity [45,46]. In line, we noted that ATG16L1 is required for PUFA-induced PTGS2/COX2 expression, which converts arachidonic acid into prostanoids with pro-inflammatory and immunomodulatory functions [45], and ATG16L1 also contributes to CXCL1 production via PTGS2/COX2 expression in our model. As such, we demonstrate that PUFA-induced ATG16L1-mediated autophagy contributes to the inflammatory tone in our model, while pleiotropic metabolic mechanisms besides the control of TLR2 activity are likely to be involved (Figure 9). Our concept is supported by previous genetic studies indicating that TLR2 signaling acts as a driver of PUFA-induced metabolic enteritis [6].

Genetic and experimental studies favor a model in which some patients with CD exhibit intestinal epithelial autophagy defects [9,16,47]. Therapeutic efficacy of rapamycin in small clinical trials (or case reports) in patients with IBD is heterogeneous [48,49] in that induction of autophagy (and associated immunosuppression) could be exploited in the future but is not an established therapy in clinical routine. However, rapamycin has evolved as an anti-aging drug currently explored for several indications in human clinical trials [50], and established immunomodulatory drugs (such as corticosteroids, azathioprine and monoclonal antibodies targeting TNF) may impact autophagy functions, collectively supporting the use of autophagy therapies in IBD [51]. Our study identifies a context-specific counterintuitive inflammatory autophagy function in IECs in the context of PUFA excess in a western diet and intestinal epithelial GPX4 deficiency in CD. As such, our study challenges our perception of protective epithelial autophagy functions in patients with IBD, which highlights the need for a better understanding of how diet affects autophagy and therapy response before we can safely harness autophagy therapies in IBD.

## Materials and methods

### Mice

*GFP-LC3* (Jackson Laboratory, strain: 027139) transgenic mice [52] were crossed with C57BL/6J *Gpx4*^*flox/WT*^;*Vil1-Cre*^*+/-*^ (*Gpx4*^*+/-IEC*^) mice [5] to obtain *GFP-LC3;Gpx4*^*flox/WT*^;*Vil1-Cre*^*-/-*^ (GFP-LC3;WT) mice and *GFP-LC3;Gpx4*^*flox/WT*^;*Vil1-Cre*^*+/-*^ (GFP-LC3;*Gpx4*^*+/-IEC*^) mice. C57BL/6J *Gpx4*^*flox/WT*^;*Vil1-Cre*^*+/-*^ (*Gpx4*^*+/-IEC*^) mice were further crossed with *atg16l1*^*flox/flox*^;* Vil1-Cre*^*+/-*^ [18] mice to obtain *Gpx4*^*flox/WT*^;*atg16l1*^*flox/flox*^;*Vil1-Cre*^*+/-*^ (*Gpx4*^*+/−IEC*^;*atg16l1*^*−/−IEC*^) mice. Experiments were performed with sex and age matched 8–10 weeks old littermates that were randomly allocated into experimental groups. For all experiments mice were housed under SPF conditions at the Medical University of Innsbruck and experimental approaches were approved as detailed under study approval.

### Mouse treatment

Mice were fed a PUFA-enriched western diet (ssniff, TD88137 + 10% fish oil [ssniff, R111F080], Table 1) [6] for three-month ad libitum. Rapamycin (1.5 mg kg^−1^ d^−1^; see Reagents below) [18] or vehicle was administered intraperitoneally once daily to WT and *Gpx4*^*+/−IEC*^ mice for the last 2 weeks of feeding the PUFA-enriched western diet. Pam2CSK4 (20 µg d^−1^) or vehicle was orally administered once daily for the last 7 days of feeding the PUFA-enriched western diet.

### Non-fasting glycemic blood measurement

Blood glucose was measured during sacrifice using an Accu-Check Performa blood glucose meter (Roche).

### Lamina propria mononuclear cell (LPMC) isolation and FACS analysis

Intestinal sections were cut open and flushed with ice-cold PBS (M&B Stricker, P04-36500) and incubated for 20 min in HBSS (Gibco, 14,175–053) with 10% FCS, 2 mM EDTA and 1 mM DTT at 37°C while shaking. Samples were washed twice with PBS and incubated for one h at 37°C in IMDM (Gibco, 21,056–023) with 20% FCS with DNAse (10 U ml^−1^; Sigma, D8764) and collagenase (128 U ml^−1^; Sigma, C1889). LPMCs were passed through a 70-µm filter and a 40-µm filter with PBS washing steps in between. Single cells were transferred into FACS buffer and incubated with an antibody cocktail (1:200, see Table 2) for immune cell characterization for 30 min at 4°C. After the incubation cells were washed and resuspended in FACS buffer and analyzed on a Cytoflex S (Beckman Coulter). Gating strategy was performed as previously described [6].

**Table 2.** FACS antibodies.

MERTK-PE-Cyanine7	Invitrogen	DS5MMER; 25–5751–82
ADGRE1/F4/80-PE-eFluor 610	Invitrogen	BM8; 61–4801–82
ITGAM/CD11b-APC-eFluor 780	Invitrogen	M1/70; 47–0112–82
ITGAX/CD11c-PE	Invitrogen	N418; 12–0114–81
PTPRC/CD45-FITC	BioLegend	30-F11; 103,107
LY6C Super Bright 600	Invitrogen	RB6-8C5; 63–5931–80
LY6G PerCP-eFluor 710	Invitrogen	1A8-Ly6g; 46–9668–80
MHC Class II (I-A/I-E)-Alexa Fluor 700	Invitrogen	M5/114.15.2; 56–5321–80
SIGLECF/CD170-eFluor 660	Invitrogen	1RNM44N;,50–1702–80
CD3-eFluor 450	Invitrogen	17A2; 48–0032–80
CD19-eFluor 450	Invitrogen	eBio1D3 (1D3); 48–0193–82
ITGA2/CD49b-eFluor 450	Invitrogen	DX5; 48–5971–82

### Murine intestinal epithelial cell isolation

Mouse intestinal epithelial cells (IECs) were isolated as previously described [18]. Briefly, intestines were flushed with ice-cold PBS and cut open. Intestinal pieces were transferred to 30 mM EDTA and repeatedly vortexed. Supernatants, containing the isolated IECs were pooled, centrifuged at 300 ×g and used for protein isolation.

### Histology

Small intestinal tissue was harvested at the end of the experiment and fixed with formalin. Dehydrated and paraffin embedded tissue was sectioned and used for hematoxylin & eosin staining for enteritis assessment. A blinded pathologist used a semi-quantitative composite scoring system, consisting of five histological subscores, multiplied by the amount of inflammation, as previously reported [18]. Briefly, the subscores are defined as followed: transmural inflammation (0: not present, 1: submucosal inflammation, 2: one focus extending into muscularis and serosa, 3: up to five foci extending into muscularis and serosa; 4: diffuse), epithelial injury (scoring from 0–3), polymorphonuclear infiltrate injury (scoring from 0–3), crypt hyperplasia injury (scoring from 0–3) and mononuclear infiltrate injury (scoring from 0–3). Scores from 0–3 are defined as 0: not present; 1: mild; 2: moderate; 3: severe.

### Detection of GFP-LC3 puncta in vivo

GFP-LC3 transgenic mice were fed a PUFA-enriched western style diet and euthanized at the end of the experiment. Small intestinal sections were embedded in OCT (Scigen, 4586) and stored at −20°C. Tissue was sectioned with at cryotome into 10-µm sections, washed with PBS and mounted with ProLong^TM^ Diamond Antifade Mountant with DAPI (Invitrogen, P36962). Imaging was performed with an Axio Observer Z1 confocal microscope (Carl Zeiss). GFP-LC3 puncta were counted in the epithelial cells of the small intestinal crypts. For better visibility of image details, all immunofluorescence images were enhanced using Adobe Lightroom 5.6.

### Detection of GFP-LC3 puncta in vitro

MODE-K IECs were seeded into chamber slides and silenced with scrambled or murine *Gpx4* siRNA according to manufacturer’s recommendation and stimulated with arachidonic acid (AA, 20 µM; Sigma, A3611) for 24 h and transfected with the Premo Autophagy Sensor LC3B-GFP (BacMam 2.0, Invitrogen, P36235). Cells were washed with PBS, fixed with formalin, and mounted with ProLong^TM^ Diamond Antifade Mountant with DAPI. Imaging was performed with an Axio Observer Z1 confocal microscope (Carl Zeiss). GFP-LC3 puncta were counted in 50 IECs. For better visibility of image details, all immunofluorescence images were enhanced using Adobe Lightroom 5.6.

### Immunoblot

Protein lysates were prepared from MODE-K IECs using M-Per (Thermo Fisher Scientific, 78,501) and from murine small intestinal isolates using T-Per (Thermo Fisher Scientific, 78,510). Bradford assay (Bio-Rad Laboratories, 5,000,006) was used to quantify protein concentration. The same amount of protein was denaturated and loaded onto SDS-PAGE, transferred to a polyvinylidene fluoride membrane (GE healthcare, GE10600023) and incubated overnight with the primary antibodies. For visualization, HRP-conjugated secondary antibodies (Cell Signaling Technology [CST], 7074) and ECL Select Western Blot Detection Reagent (Amersham, RPN2235) was used. Densitometry was performed with ImageJ. Full immunoblots of Figures 1D and 2A are shown in Data S1.

Anti-GPX4 (Abcam, ab125066), Anti-LC3B (Cell Signaling Technology [CST], 2775), anti-HSPA5/GRP78 (CST, 3177), anti-ATG16L1 (MBL, M150-3), anti-phospho-MAPK/SAPK/JNK (CST, 4668) anti-MAPK/SAPK/JNK (CST, 9265), anti-phospho-JUN/c-Jun (CST, 2361), anti-JUN/c-Jun (CST, 9165), anti-phospho-ERN1/IRE1α (Abcam, ab48187), anti-ERN1/IRE1α (CST, 3294), anti-ATG5 (CST, 12,994), anti- ATG7 (CST, 8558), anti-ATG12 (CST, 4180), anti-SQSTM1/p62 (CST, 5114) anti-phospho-MAPK/p38 (CST, 4888), anti-MAPK/p38 (CST, 9212), anti-cleaved CASP3 (CST, 9664), anti-PTGS2/COX2 (CST, 12,282), anti-GPX1 (Abcam ab22604), anti-GPX2 (Abcam ab137431), anti-SOD1 (Abcam, ab13498), anti-PRDX6 (Abcam, ab59543) and anti-GAPDH (CST, 14C10).

### Rna extraction and qRT-PCR

RNA was extracted from MODE-K IECs using the RNeasy mini kit (Qiagen, 74,104). cDNA synthesis was performed with M-MLV reverse transcriptase (Invitrogen, 28,025,013). qPCR was performed with a MESA GREEN qPCR MasterMix (Eurogentec, RT-SY2X-06+WOULR) on a QuantStudio 3 (Thermo Fisher Scientific). qPCR protocol included one denaturation step at 95°C for 10 min, followed by 40 cycles of 15 s denaturation at 95°C, 30 s of Primer annealing and 30 seconds elongation at 72°C. Melting curve analysis was performed at the end of the protocol to test for PCR specificity. mRNA expression was calculated by using the 2^−ΔCT^ method. *Actb/ß-actin* was used as housekeeping gene. Primers are listed in Table 3 and were obtained from Primerbank [53].

**Table 3.** Primer used for qPCR.

mouse	Forward	Reverse
*Actb*	GATGCTCCCCGGGCTGTATT	GGGGTACTTCAGGGTCAG GA
*Gpx4*	GATGGAGCCCATTCCTGAACC	CCCTGTACTTATCCAGGCAGA
*Atg16l1*	TGTGAGAGTCCCAACTACTGC	TCCCAAAGTTTCACCCTGCG
*Atg5*	AGCCAGGTGATGATTCACGG	GGCTGGGGGACAATGCTAA
*Atg7*	GTTCGCCCCCTTTAATAGTGC	TGAATCCCAACGTCAAGCGG
*Atg12*	GTCGCCTCGGAAGTTGTTTA	ATCCCCATGCCTGGGATTTG
*Ncoa4*	GAACCATCAGGACACATGGAAA	AGGAGCCATAGCCTTGGGT
*Rab7a*	AGGCTTGGTGCTACAGGAAAA	CTTGGCCCGGTCATTCTTGT
*Cxcl1*	CTGGGATTCACCTCAAGAACATC	CAGGGTCAAGGCAAGCCTC
*Ptgs2*	TGCACTATGGTTACAAAAGCTGG	TCAGGAAGCTCCTTATTTCCCTT
*Map1lc3b*	TTATAGAGCGATACAAGGGGGAG	CGCCGTCTGATTATCTTGATGAG
*Vps4a*	GATCTGGTGACAAAAGCCACA	CTTTGCTCGAATGCTCTCCTT
*Spart*	ATCCCTGGGAGATCAAGTCAC	CTGCCTTGGTCTATTGAGGAAC
*Rb1cc1*	GACACTGAGCTAACTGTGCAA	GCGCTGTAAGTACACACTCTTC
*Becn1*	ATGGAGGGGTCTAAGGCGTC	TCCTCTCCTGAGTTAGCCTCT

### Cell culture

MODE-K (*Mus musculus*) small intestinal epithelial cells (provided by D. Kaiserlian [54]), MODE-K *atg16l1*^*-/-*^, MODE-K *atg16l1*^*-/-*^ *ATG16L1WT* and MODE-K *atg16l1*^*-/-*^ *ATG16L1*^*T300A*^ were cultivated in high-glucose DMEM (Biowest, MS01BJ1007) with 10% FCS (Sigma, S0615), 10 mM HEPES (PAN Biotech, P05-01100), 1 mM non-essential amino acids (Gibco, 11,140–050), 100 U ml^−1^ penicillin and 100 µg ml^−1^ streptomycin (PAN Biotech, P06-07050). HEK-Blue^TM^ mTLR2 cells (InvivoGen, hkbmtlr2) were cultivated in high-glucose DMEM (Biowest, MS01BJ1007) with 10% FCS (Sigma, S0615), 1x HEK Blue Selection (InvivoGen, hb-sel), 100 U ml^−1^ penicillin, 100 µg ml^−1^ streptomycin and 100 µg ml^−1^ normocin (InvivoGen, ant-nr).

### Reagents

The following reagents were used: arachidonic acid (AA, 20 µM; Sigma, A3611), stearidonic acid (SDA, 50 µM; Sigma, SMB00291), rapamycin (Rapa, 500 nM; Enzo, A275-0005), bafilomycin A_1_ (100 nM; InvivoGen, tlrl-baf1), chloroquine (50 µM; Sigma, C6628), oxPAPC (100 µg/ml; Avanti Polar Lipids, 870604P), 4-HNE (100 µM; Sigma, 393,204), TNF (50 ng ml^−1^; Peprotech, 315-01A), cycloheximide (CHX, 10 mg ml^−1^; Sigma, 239,765), Pam2CSK4 (0,1 µg ml^−1^; Invivogen, tlrl-pm2s-1), celecoxib (CEB, 0.1 µM, 1 µM, 10 µM; Tocris, 3786/10).

### siRNA silencing

MODE-K, MODE-K *atg16l1*^*-/-*^, MODE-K *atg16l1*^*-/-*^ ATG16L1WT and MODE-K *atg16l1*^*-/-*^ *ATG16L1*^T300A^ cells were seeded in 12-well plates and silenced according to manufacturers recommended protocol for 48 h with RNAiMAX (Thermo Fisher Scientific,13778100). The following siRNAs were used: *Gpx4* siRNA (*siGpx4*; Ambion, s122098), scrambled control siRNA (siCtrl; Ambion 4,390,843), *Atg16l1* (*siAtg16l1*; Ambion, s94890, s94891), human *ATG16L1* (*siATG16l1*; Ambion, 30,069), *Atg5* (*siAtg5;* Ambion, s62452), *Atg7* (*siAtg7*; Ambion, s92536), *Atg12* (*siAtg12*, Ambion, s85218), *Ncoa4* (*siNcoa4*; Ambion, s77518) and *Rab7a* (*siRab7a*, Ambion, s232059), *Vps4a* (*siVps4a*; Ambion, s100254), *Spart* (*siSpart*; Ambion, s106133), *Rb1cc1* (*siFip200*; Ambion, s63492), *Becn1* (*siBecn1*; Ambion, s80166), *Map1lc3b* (*siMap1lc3b*; Ambion, s85061).

### CRISPR-Cas9 gene editing

Gene disruption of *Atg16l1* in MODE-K IECs was performed by using the CRISPR-Cas9 system [55] as previously described [5]. The guide RNA for *atg16l1*^*-/-*^ IECS was TGCAAGCCGAATCTGGACTG targeting exon 12. Transfection was performed for 48 h and positive cell clones were selected with puromycin (2 µg ml^−1^; Gibco, A1113803).

### Generation of MODE-K atg16l1^−/−^ ATG16L1 WT and MODE-K atg16l1^−/−^ ATG16L1^T300A^

For the generation of the MODE-K *atg16l1*^*-/-*^ *ATG16L1WT* and MODE-K *atg16l1*^*-/-*^ *ATG16L1*^*T300A*^ WT and T300A variant of human ATG16L1 coding sequence was synthesized by IDT as a double-stranded DNA fragment (gBlock) with inserted single nucleotide exchange from ACT (threonine/T) to GCT (alanine/A). Both variants were inserted via Gateway LR reaction into the backbone of plasmid pPB-GW-Hygro (modified from Addgene, 83,961; deposited by Eleanor Chen, http://n2t.net/addgene:83961; RRID:Addgene_83961). The resultant plasmids were isolated, validated by sanger sequencing and subsequently used for transfection of *MODE-K atg16l1*^*-/-*^ cells using the piggyback transposon system. Cells were treated with hygromycin (400 µg ml^−1^; Sigma 10,843,555,001) for clone selection. Graphical illustration of the generation of MODE-K *atg16l1*^*-/-*^ *ATG16L1WT* and MODE-K *atg16l1*^*-/-*^ *ATG16L1*^*T300A*^ cell clones is depicted in **Fig. S4A, B.**

### In-vitro ATG16L1 cleavage assay

ATG16L1 cleavage assay was performed as previously described [9]. Briefly MODE-K *atg16l1*^*-/-*^ ATG16L1WT and MODE-K *atg16l1*^*-/-*^ *ATG16L1*^*T300A*^ were stimulated with TNF (50 ng ml^−1^;), CHX (10 mg ml^−1^; Sigma, 239,765) for 3 h and protein was isolated and equal amounts of protein was used for the immunoblot to represent ATG16L1 cleavage.

### TLR2 activity assay

HEK-Blue^TM^ mTlr2 cells (InvivoGen, hkb-mtlr2) were used to assess TLR2 activity by monitoring AP-1 activity. Assay was performed according to manufacturers’ recommendation. HEK-Blue^TM^ mTlr2 cells were incubated with potential TLR2 agonists at 37°C and 5% CO_2_ for up to 16 h. AP-1 activity was determined by measuring SEAP production with a photometer at 620 nm.

### Lipid peroxidation detection by flow cytometry

MODE-K IECs were harvested and incubated with BODIPY581/591 C11 for 30 min at 37°C in FACS buffer and then analyzed on a Cytoflex S (Beckman Coulter). DAPI was used to distinguish between viable and dead cells.

### RNA Sequencing

IECs were seeded in 12-well plates and silenced with *Gpx4* siRNA (*siGpx4*), *Atg16l1* siRNA (*siAtg16l1*) or scrambled control siRNA (siCtrl) according to manufacturer’s recommendation for 48 h and treated with AA (20 µM) or SDA (50 µM) for 8 h. RNA was extracted using the RNeasy mini kit (Qiagen 74,104). Purified total-RNA was submitted to transciptome analysis at the Medical University Innsbruck MultiOmics Sequencing Core for the purpose of gene-expression profiling, using the QuantSeq 3′ mRNA-Seq Library Prep method (Lexogen, Vienna Biocenter). Quality-validated, barcoded libraries were multiplexed and sequenced using Illumina NovaSeq technology.

### Extraction and analysis of phospholipids by UPLC-MS/MS

MODE-K WT and *atg16l1*^*-/-*^ IECs were seeded in 6 well plates and silenced with *Gpx4* siRNA (*siGpx4*) or scrambled control siRNA (siCtrl) according to manufacturer’s recommendation for 48 h and treated with AA (20 µM) or SDA (50 µM) in DMEM medium containing 1% FCS for 30 min. Phospholipids were extracted from cell pellets by the sequential addition of PBS pH 7.4, methanol (spiked with internal standards), chloroform, and saline (final ratio 14:34:35:17). The chloroform layer was recovered and evaporated using an Eppendorf Concentrator Plus system (Hamburg, Germany; nonpolar phase: high vapor pressure application mode), stored at −20°C, and dissolved in methanol prior to ultra-high-performance liquid chromatography-mass spectrometry (UPLC-MS/MS) analysis [56,57]. 1-Pentadecanoyl-2-oleoyl(d7)-sn-glycero-3-phosphocholine (PC[15:0/-18:1-d7], Avanti Polar Lipids, Alabaster, AL/Sigma-Aldrich, 791637C) and 1-pentadecanoyl-2-oleoyl(d7)-sn-glycero-3-phosphoethanolamine (PE[15:0_18:1-d7], Avanti Polar Lipids/Sigma-Aldrich, 791638C)] were used as internal standards.

PC and PE were separated on an Acquity UPLC BEH C8 column (130 Å, 1.7 μm, 2.1 × 100 mm, Waters, Milford, MA) using an ExionLC AD UHPLC system (Sciex, Framingham, MA). The gradient containing mobile phase A (water:acetonitrile 90:10, 2 mM ammonium acetate) and B (water: acetonitrile 5:95, 2 mM ammonium acetate) was ramped from 75 to 85% B over 5 min and then further increased to 100% B within 2 min, which was maintained isocratically for another 2 min. The flow rate was set to 0.75 ml/min and the column temperature to 45°C. PC and PE species were detected by a QTRAP 6500+ mass spectrometer (Sciex) equipped with an IonDrive Turbo V Ion Source and a TurboIonSpray probe for electrospray ionization [57]. Optimized mass spectrometry parameters were as follows: curtain gas, 40 psi; collision gas, medium; ion spray voltage, −4500 V; heated capillary temperature, 350°C (for PC), 650°C (for PE); sheath gas pressure, 55 psi; auxiliary gas, 75 psi; declustering potential, −44 V (for PC) and −50 V (for PE); entrance potential, −10 V; collision energy, −46 eV (for PC) and −38 eV (for PE); collision cell exit potential, −11 V (for PC) and −12 V (for PE). The QTRAP 6500+ system was operated in negative ionization mode using scheduled multiple reaction monitoring (MRM).

For the detection of PE and PC species, the transitions from [M-H]^−^ to both fatty acid anions were monitored [56,57]. The instruments were operated by Analyst 1.7.1 (Sciex), and the mass spectra were processed by Analyst 1.6.3 (Sciex).

For the quantitative analysis of PE and PC species, combined peak areas from the transitions to the two fatty acid anions were calculated. Relative intensities of individual phospholipid species or subfractions are given as percentage of all species detected in the corresponding phospholipid subclass ( = 100%). To calculate absolute lipid quantities, signals were normalized to a subgroup-specific internal standard [PE(15:0_18:1-d7) or PC[15:0_18:1-d7]. The fractions of saturated fatty acids (SFA), monounsaturated fatty acids (MUFA) and PUFA (≥2 double bounds) in phospholipids are calculated from mean signal intensities that are derived by two, and the proportions are then distributed according to the sn-1 or sn-2 fatty acid to either fraction.

### Extraction and analysis of lipid mediators by UPLC-MS/MS

MODE-K WT and *atg16l1*^*-/-*^ IECs were seeded in 6 well plates and silenced with *Gpx4* siRNA (*siGpx4*) or scrambled control siRNA (siCtrl) according to manufacturer’s recommendation for 48 h and treated with AA (20 µM) or SDA (50 µM) in DMEM medium containing 1% FCS for 30 min. The collected cell supernatants (1 ml) and the medium control (DMEM medium containing 1% FCS) were transferred to 2 ml of ice-cold methanol containing deuterium-labeled internal standards (d_4_-PGE_2_, 314,010, d_5_-RvD_2_, 11,184, d_8_-5(S)-HETE, 334,230, d_4_-LTB_4_, 320,110, d_5_-LXA4, 10,007,737, d_11_-8,9-EpETE, 10,009,532, d_8_-2-AG, 362,160, d_8_-AEA, 390,050, 200 pg each; and d_8_-AA, 390,010, 2000 pg; Cayman Chemical) to allow quantification of lipid mediators (LM) and assess sample recovery. Lipid mediators were extracted by solid phase extraction using Sep-Pak® Vac 6cc (500 mg 6 ml^−1^ C18; Waters, WAT043395) as previously described [58,59]. Briefly, samples were kept at −20°C for at least 60 min to allow protein precipitation. After centrifugation (750 × g, 4°C, 10 min), samples were mixed with 8 ml acidified water (pH = 3.5) and loaded onto solid phase cartridges that were pre-equilibrated with 6 ml methanol and 2 ml water. After washing with 6 ml water and 6 ml ice-cold n-hexane, LM were eluted with 6 ml methyl formate. Finally, the recovered samples were evaporated to dryness using a TurboVap LV evaporation system (Biotage, Uppsala, Sweden) and resuspended in 100 µl methanol-water (50:50, v/v) and centrifuged twice (21,100 × g, 4°C, 5 min) before subjection to UPLC-MS/MS analysis.

Chromatographic separation of LM was carried out at 55°C on an Acquity UPLC BEH C-18 column (130Å, 1.7 μm, 2.1 × 100 mm; Waters, 186,002,352) using an ExionLC AD UHPLC system (Sciex, Framingham, MA, USA). The gradient of mobile phase A (water:MeOH, 90:10, 0.01% acetic acid) and mobile phase B (MeOH, 0.01% acetic acid) was ramped at a flow rate of 0.35 ml min^−1^ from 35.6% to 84.4% B within 12.5 min, then to 87.0% B within 2.5 min, followed by 3 min of isocratic elution at 97.8% B, and at the end switched to 35.6% B [60]. LM were detected by a QTRAP 6500+ mass spectrometer (Sciex) equipped with an IonDrive Turbo V Ion Source and a TurboIonSpray probe for electrospray ionization. The QTRAP 6500+ system was operated in negative ionization mode using scheduled multiple reaction monitoring (MRM, detection window: 120 s) and switched to positive ion mode for detection of endocannabinoids. The curtain gas was set to 40 psi, the collision gas to medium, the ion spray voltage to −4000 V, the heated capillary temperature to 500°C, and the sheath and auxiliary gas pressure to 40 psi. Transitions selected for quantitation and the corresponding declustering potential (DP), entrance potential (EP), collision energy (CE) and collision cell exit potential (CXP) are listed in Table 4. Mass spectra were acquired and processed using Analyst 1.7.1 (Sciex) and Analyst 1.6.3 (Sciex), respectively. The retention time and diagnostic ions for each LM were confirmed by means of commercially available external standards (Cayman Chemical/Biomol). Absolute lipid quantities refer to an external standard calibration and were normalized to a subclass-specific deuterated internal standard as well as cell numbers.

**Table 4.** Quantitative MRM transitions for the UPLC-MS/MS analysis.

Q1	Q3	ID	DP (V)	EP (V)	CE (eV)	CXP (V)	Q1	Q3	ID	DP (V)	EP (V)	CE (eV)	CXP (V)
327	116	d8-5S-HETE	−80	−10	−17	−10	319	219	15-HETE	−80	−10	−19	−12
339	197	d4-LTB_4_	−80	−10	−22	−13	319	179	12-HETE	−80	−10	−21	−12
355	193	d4-PGE_2_	−80	−10	−25	−16	319	167	11-HETE	−80	−10	−21	−12
356	115	d5-LXA4	−80	−10	−19	−14	319	115	5-HETE	−80	−10	−21	−12
380	141	d5-RvD2	−80	−10	−23	−14	295	171	9-HODE	−60	−10	−19	−13
311	267	d8-AA	−100	−10	−16	−18	295	195	13-HODE	−60	−10	−25	−13
351	235	LXA4	−80	−10	−20	−13	279	163	12-HHT	−30	−10	−30	−13
351	189	PGD_2_	−120	−10	−20	−13	335	115	5S,6 R-diHETE	−80	−10	−20	−13
351	271	PGE_2_	−120	−10	−20	−13	335	235	5,15-diHETE	−80	−10	−22	−13
351	233	PGD_2_	−80	−10	−16	−15	303	259	AA	−100	−10	−16	−18
353	193	PGF_2α_	−80	−10	−34	−11	301	257	EPA	−100	−10	−16	−18
369	169	TXB_2_	−80	−10	−22	−15	327	283	DHA	−100	−10	−16	−18
353	317	PGE_1_	−90	−10	−18	−15	329	285	DPA	−100	−10	−16	−18
359	153	PDX	−80	−10	−21	−9	277	233	ALA	−140	−10	−20	−12
359	199	RvD5	−80	−10	−21	−13	330	155	d11-8.9-EET	−30	−5	−20	−10
359	250	Maresin 1	−80	−10	−20	−16	319	167	8.9-EET	−40	−5	−20	−10
359	221	Maresin 2	−80	−10	−20	−12	319	167	11.12-EET	−40	−10	−20	−10
333	199	RvE2	−80	−10	−24	−17	319	219	14.15-EET	−60	−10	−20	−10
333	115	RvE4	−80	−10	−22	−13	337	145	5.6-DHET	−70	−5	−20	−10
335	195	LTB_4_ isomers	−80	−10	−22	−13	337	127	8.9-DHET	−60	−5	−30	−15
335	195	LTB_4_	−80	−10	−22	−13	337	167	11.12-DHET	−30	−5	−30	−15
343	255	21-HDHA	−80	−10	−17	−14	337	207	14.15-DHET	−60	−5	−20	−10
343	245	17-HDHA	−80	−10	−17	−14	343	153	10.11-EpDPA	−40	−10	−20	−13
343	205	14-HDHA	−80	−10	−17	−14	343	233	16.17-EpDPA	−20	−5	−15	−20
343	193	13-HDHA	−80	−10	−17	−14	343	241	19.20-EpDPA	−40	−10	−20	−13
343	141	7-HDHA	−80	−10	−18	−15	317	167	11.12-EpETE	−40	−10	−15	−15
343	101	4-HDHA	−80	−10	−17	−15	317	207	14.15-EpETE	−30	−10	−20	−10
345	247	17-HDPA	−80	−10	−17	−14	317	215	17.18-EpETE	−40	−5	−15	−10
345	207	14-HDPA	−80	−10	−17	−14	356	63.2	d8-AEA	30	10	20	10
345	195	13-HDPA	−80	−10	−17	−14	387	294	d8-2-AG	50	10	20	20
345	143	7-HDPA	−80	−10	−18	−15	348	287	AEA	20	10	20	15
317	259	18-HEPE	−80	−10	−16	−23	300	62	PEA	30	10	20	10
317	219	15-HEPE	−80	−10	−18	−12	327	62.1	OEA	30	10	20	40
317	179	12-HEPE	−80	−10	−19	−12	379	287	2-AG	40	10	20	15
317	167	11-HEPE	−80	−10	−19	−12	379	287	1-AG	30	10	20	15
317	115	5-HEPE	−80	−10	−18	−12							

AA, arachidonic acid; AEA: anandamide; AG: arachidonoylglycerol; ALA: α-linolenic acid; AT, aspirin-triggered; EET, epoxyeicosatrienoic acid; EPA, eicosapentaenoic acid; DHA, docosahexaenoic acid; DHET, dihydroxyeicosatrienoic acid; DPA, docosapentaenoic acid; EPA: eicosapentaenoic acid; EpDPA: epox docosapentaenoic acid; EpETE: epox eicosatetraenoic acid; (di)HDHA, (di)hydroxydocosahexaenoic acid; HDPA: hydroxydocosapentaenoic acid; HEPE, hydroxyeicosapentaenoic acid; (di)HETE, (di)hydroxyeicosatetraenoic acid; HODE: hydroxyoctadecadienoic acid; LT: leukotriene; LX: lipoxin; OEA: oleoylethanolamide; PD, protectin; PEA: palmitoylethanolamide; PG: prostaglandin; Rv: resolvin; TX, thromboxane.

### Enzyme linked immunosorbent assay (ELISA)

Cytokines were detected in cell culture supernatants and mouse serum using murine CXCL1/KG ELISA (R&D, DY453) and murine IL6 ELISA (BD Biosciences, 555,240). Cell supernatants were collected and centrifuged at 300 × g for 10 min. Heparinized murine blood was centrifuged at 2000 × g for 10 min and serum was stored at −80°C. The assays were performed according to manufacturer’s recommendation.

### Glutathione GSH/GSSG Assay Kit

GSH:GSSG ratio was detected in cell lysates of siCtrl and *siGpx4* IECs using Glutathione GSH/GSSG Assay Kit (Sigma, MAK440) according to the manufacturer’s recommendations.

### Immunohistochemistry

Formalin-fixed paraffin-embedded sections were deparaffinized and rehydrated in descending ethanol. Antigen retrieval was performed for 15 min in subboiling citrate buffer and peroxidase activity was blocked. Slides were incubated with the primary antibody overnight at 4°C. Immunoreactivity was visualized by horseradish (HRP)-driven 3,3 diaminobenzidine (DAB) turnover of the HRP-labeled secondary antibody. LYZ (lysozyme)-positive cells were quantified in 50 consecutive crypts.

Anti-LYZ/lysozyme (Abcam, ab108508), anti-MPO (Dako GA511), anti-CD4 (Dako IR649), anti-CD8 (Dako GA623).

### RNAseq data analysis

We used the nf-core/rnaseq (version 3.11.1) pipeline to align the raw reads to the mouse genome (GRCm39) with STAR and to assess the read counts on the gene models from GENCODE version M31 with Salmon [61–63]. We ran the pipeline with the default parameters except for generating the STAR index, where we used the `–sjdbOverhang 200` parameter, and the gene quantification with Salmon where we set the following parameters “–noLengthCorrection`, which accounts for the Lexogen 3” QuantSeq RNA sequencing library. Differential expressed genes were calculated using DESeq2 (version 1.42.1) using a FDR < 0.1 after Independent Hypothesis Weighting (IHW) as significance cutoff [64]. Heatmaps of differential expressed genes and GSEA plots were generated using the R packages ComplexHeatmap (version 2.18.0), ClusterProfiler (version 4.10.1) and enrichplot (version 1.22.0) [65]. Genesets for the AA metabolism, the MAPK signaling pathway and the protein processing in endoplasmic reticulum were extracted from the KEGG [66] database.

### Statistical analysis

Data are expressed as mean ± standard error of the mean (SEM) unless otherwise stated. Statistical analysis was performed with GraphPad Prism 10. Outlier testing was performed in parametric sample sets. If not otherwise indicated statistical significance was assessed with an unpaired Students T-Test or a one-way ANOVA with Bonferroni correction: *p < 0.05, **p < 0.01, ***p < 0.001, ****p < 0.0001.

## Study approval

Animal experiments were performed in accordance with institutional guidelines of the Medical University of Innsbruck and following approval by federal authorities (2020–0.827.349, 2020–0.398.000, 2022–0.892.260 and 2024–0.649.853).

## Supplementary Material

Data S1 R3 (1)...docx

Supplementary Figures ATG16L1 enteritis R6v2...docx

## Data Availability

Data generated from RNA Sequencing are available upon request.
