# Supplementary material for: Chronic enteritis triggered by diet westernization is driven by epithelial ATG16L1-mediated autophagy
Source: Autophagy. 2026 Jan 5;22(2):391–408. doi: 10.1080/15548627.2025.2600906 (PMC12834163; doi:10.1080/15548627.2025.2600906)
Supplement: Data S1 R3 (1)...docx [file KAUP_A_2600906_SM4698.docx]

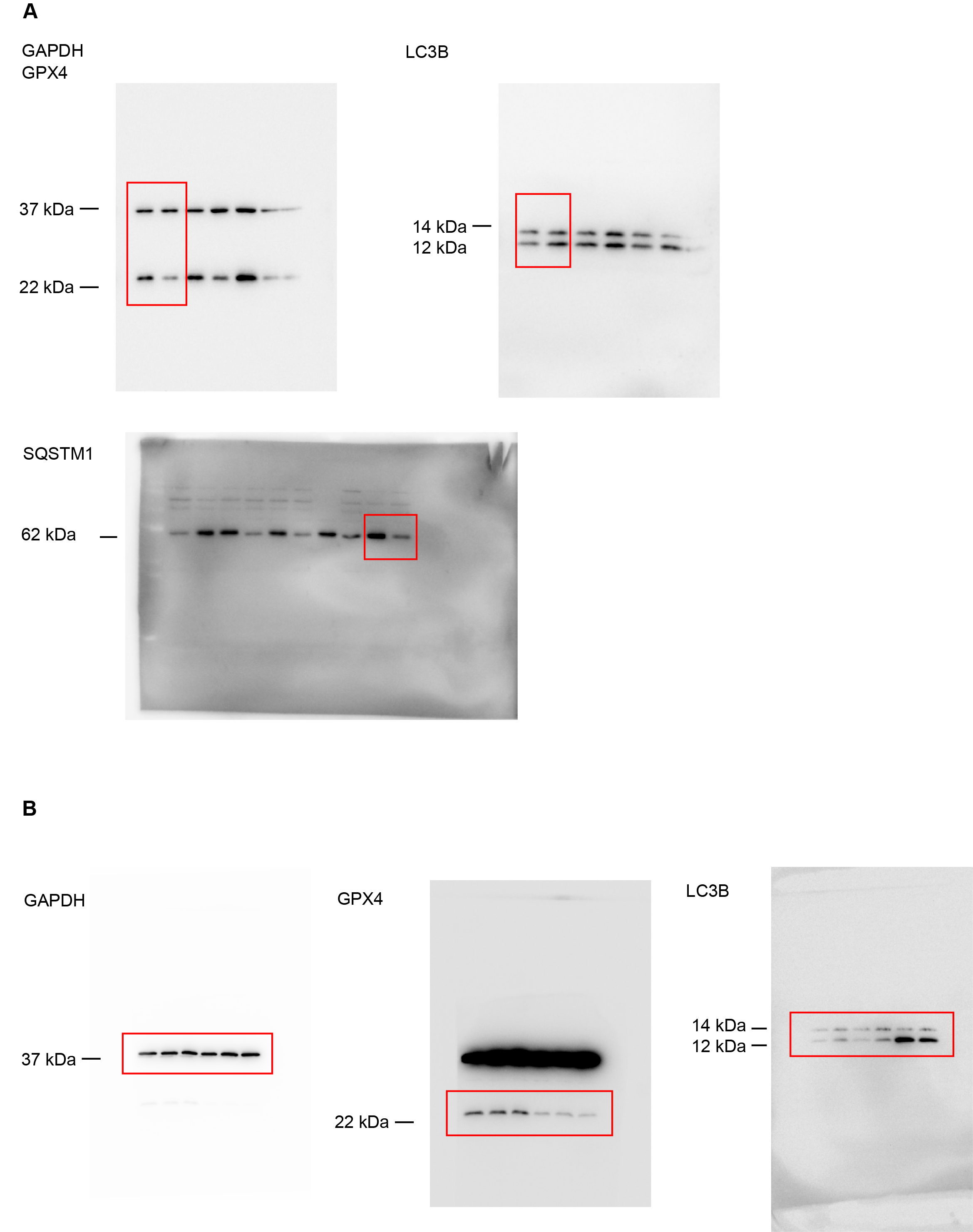


**Data S1.** Full immunoblots. **(A, B**) uncropped immunoblots from Figure 1D (**A**) and Figure 2A (**B**). Target proteins and kDa are indicated. [Change labels to “LC3B”.]
