# Supplementary material for: Chronic enteritis triggered by diet westernization is driven by epithelial ATG16L1-mediated autophagy
Source: Autophagy. 2026 Jan 5;22(2):391–408. doi: 10.1080/15548627.2025.2600906 (PMC12834163; doi:10.1080/15548627.2025.2600906)
Supplement: Supplementary Figures ATG16L1 enteritis R6v2...docx [file KAUP_A_2600906_SM4697.docx]

Supplementary Figures


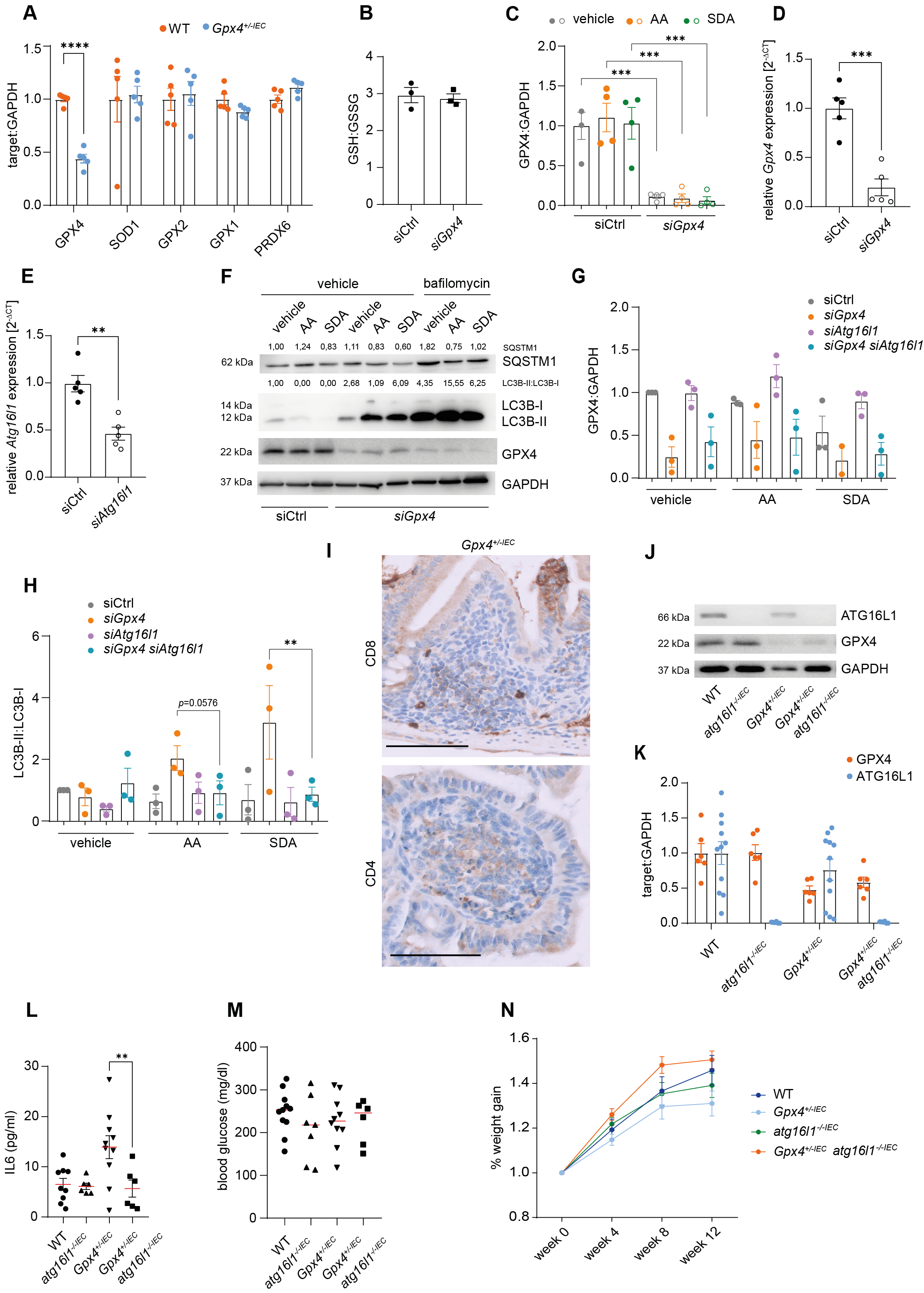


**Figure S1.** PUFAs trigger inflammation in *Gpx4*-deficient intestinal epithelium. (**A**) Quantification of GPX4, SOD1, GPX2, GPX1 and PRDX6 compared to GAPDH of intestinal epithelial scrapings of WT and *Gpx4^+/−IEC^* mice after three months exposure to a PUFA-enriched Western diet (*n* = 5). GAPDH served as the loading control. A representative immunoblot is shown in Figure 1G. (**B**) GSH:GSSG ratio of siCtrl and *siGpx4* IECs (*n* = 3). (**C**) Quantification of GPX4 compared to GAPDH of siCtrl and *siGpx4* IECs stimulated with ω-6 PUFA (AA) or ω-3 PUFA (SDA) for 24 h. (*n* = 4). GAPDH served as a loading control. A representative immunoblot is shown in Figure 2 A. (**D, E**) Expression of *Gpx4* (**D**) and *Atg16l1* (**E**) relative to *Actb* in silenced MODE-K IECs determined by qPCR (*n* = 5). (**F**) Immunoblot of GPX4, LC3B-I:II and SQSTM1 of siCtrl and *siGpx4* IECs stimulated with ω-6 PUFA (AA) or ω-3 PUFA (SDA) for 24 h with or without bafilomycin A_1_ treatment. (*n* = 1). (**G, H**) Quantification of GPX4 (**G**) and LC3B-II:I (**H**) of siCtrl, *siGpx4, siAtg16l1* and *siGpx4 siAtg16l1* IECs after ω-6 PUFA (AA) or ω-3 PUFA (SDA) stimulation for 24 h compared to GAPDH. (*n* = 3). A representative immunoblot is shown in Figure 2E**.** (**I**) Representative images of CD4^+^ and CD8^+^ T cells of inflamed mucosa of *Gpx4^+/−IEC^* mice after three months exposure to a PUFA-enriched Western diet. Note that these are higher magnifications from Figure 3 D, E. Scale bar: 100 µm. (**J, K**) A representative immunoblot (**J**) and quantification (**K**) of ATG16L1 and GPX4 of WT, *Gpx4^+/−IEC^, atg16l1^-/-IEC^* and *Gpx4^+/−IEC^;atg16l1^-/-IEC^* mice after three months exposure to a PUFA-enriched Western diet. GAPDH served as a loading control (*n* ≥ 5). (**L**) Quantification of IL6 in the serum of WT, *Gpx4^+/−IEC^, atg16l1^-/-IEC^* and *Gpx4^+/−IEC^; atg16l1^-/-IEC^* mice after three months exposure to a PUFA-enriched Western diet. (*n =* 5/10). (**M**) Non-fasted blood glucose levels of WT, *Gpx4^+/−IEC^, atg16l1^-/-IEC^* and *Gpx4^+/−IEC^; atg16l1^-/-IEC^* mice after three months of PUFA WD. (*n* ≥ 6). Median is shown. (**N**) Relative weight gain of WT, *Gpx4^+/−IEC^, atg16l1^-/-IEC^* and *Gpx4^+/−IEC^; atg16l1^-/-IEC^* mice after three months exposure to a PUFA-enriched Western diet. (*n* > 8). **P<0.05,* ***P<0.01,* ****P<0.001,* *****P<0.0001.*


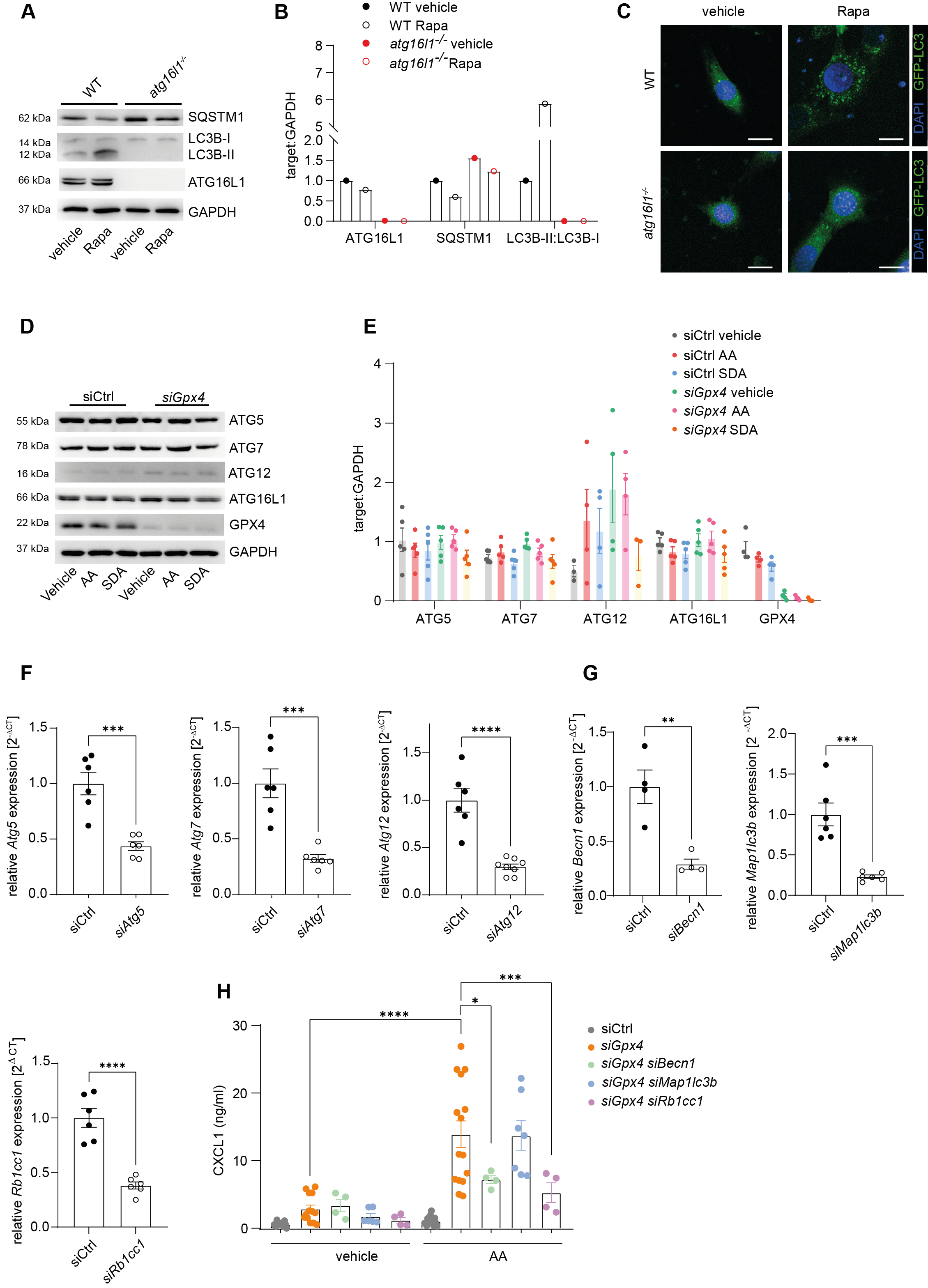


**Figure S2.** Autophagy deficiency blocks PUFA-induced CXCL1 production. (**A, B**) A representative immunoblot (**A**) and quantification (**B**) of ATG16L1, LC3B-II:I and SQSTM1 of WT and CRISPR-Cas9 edited MODE-K (*atg16l1^-/-^*) stimulated with rapamycin (Rapa) or vehicle. GAPDH served as the loading control. (*n* = 1) (**C**) Representative confocal images of GFP-LC3 puncta (green) of WT and CRISPR-Cas9-edited MODE-K (*atg16l1^-/-^*) stimulated with rapamycin (Rapa) or vehicle. DAPI (blue) indicates nuclei. Scale bar: 5 µm. (**D, F**) A representative immunoblot (**D**) and quantification (**F**) of ATG16L1, ATG12, ATG7, ATG5 and GPX4 of siCtrl and *siGpx4* IECs stimulated with ω-6 PUFA (AA) or ω-3 PUFA (SDA) for 24 h. (*n* = 4-5). GAPDH served as a loading control. (**F**) Relative expression of *Atg5*, *Atg7* and *Atg12* relative to *Actb* in silenced MODE-K IECs determined by qPCR (*n* > 3). (**G**) Relative expression of *Becn1,* *Map1lc3b* and *Rb1cc1/Fip200* relative to *Actb* in silenced MODE-K IECs determined by qPCR (*n* > 4). (**H**) Quantification of CXCL1 in the supernatant of siCtrl, *siGpx4,* *siGpx4 siBecn1*, *siGpx4 siMap1lc3b* and *siGpx4 siRb1cc1* after ω-6 PUFA (AA) stimulation for 24 h. (*n* ≥ 4). Please not that siCtrl and *siGpx4* are identical as in **Fig. S3C** as experiments were performed at the same time. **P<0.05,* ***P<0.01*, ****P<0.001,* *****P<0.0001*.


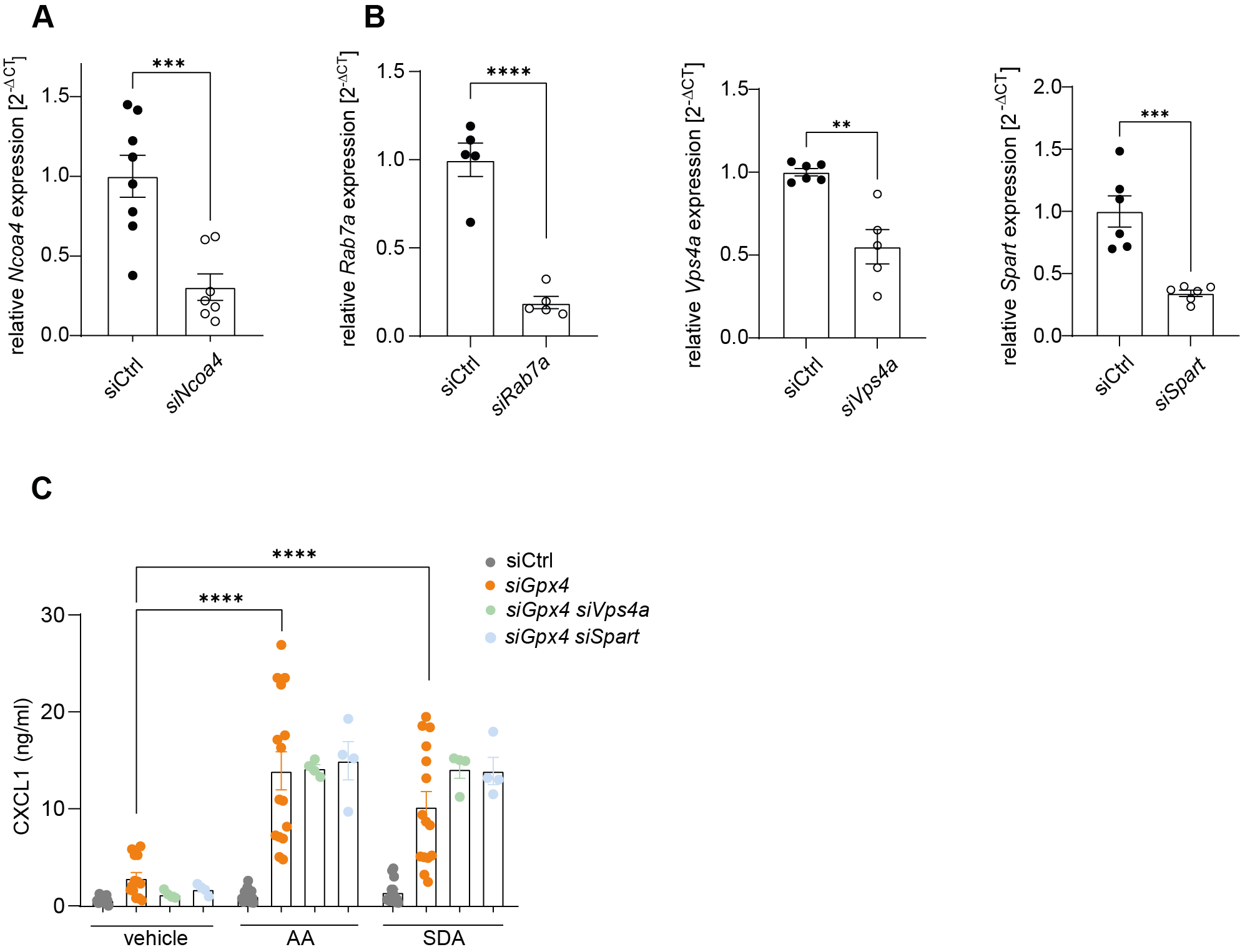


**Figure S3.** *Ncoa4* and lipophagy receptors are dispensable for PUFA-induced CXCL1 production. (**A**) Relative expression of *Ncoa4* relative to *Actb* in silenced MODE-K IECs determined by qPCR (*n* > 3). (**B**) Relative expression of *Rab7a, Vps4a* and *Spart/Spg20* relative to *Actb* in silenced MODE-K IECs determined by qPCR (*n* > 3). (**C**) Quantification of CXCL1 in the supernatant of siCtrl, *siGpx4,* *siGpx4 siVps4a* and *siGpx4 siSpart* after ω-6 PUFA (AA) or ω-3 PUFA (SDA) stimulation for 24 h. (*n* ≥ 4). Please note that sictrl and *siGpx4* are identical as in **Fig. S2H** as experiments were performed at the same time. ***P<0.01*, ****P<0.001,* *****P<0.0001*.


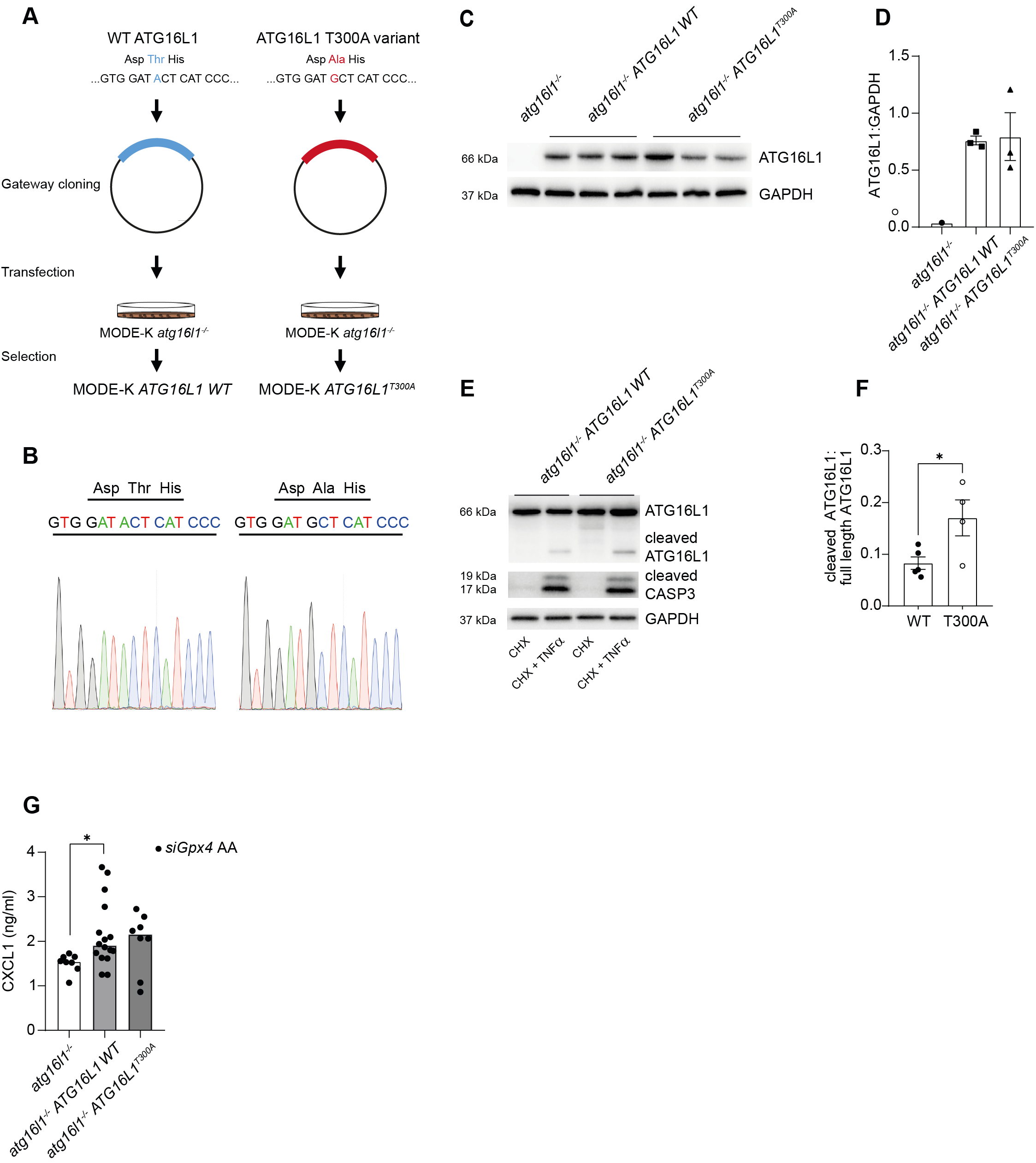


**Figure S4.** The human T300A CD risk variant is not impacting PUFA-induced CXCL1 production in IECs. (**A**) Schematic model of the generation of *ATG16L1* *WT* and *ATG16L1^T300A^* expressing *atg16l1^-/-^* IECs. WT variant is depicted in blue, while T300A (Thr to Ala) nucleotide change is depicted in red. DNA fragments were inserted into plasmids via gateway cloning and transfection of *atg16l1^-/-^* IECs was performed with the piggyback transposon system. Details are provided in the material and methods section. (**B**) Sanger sequencing confirmed the targeted mutation (ACT -> GCT). Representative chromogram is shown. (**C, D**) A representative immunoblot (**C**) and quantification (**D**) of ATG16L1 of *atg16l1^-/-^, atg16l1^-/-^ATG16L1 WT* and *atg16l1^-/-^ ATG16L1^T300A^* IECs. GAPDH served as the loading control. (**E, F**) A representative immunoblot (**E**) and quantification (**F**) of ATG16L1, cleaved ATG16L1 and cleaved CASP3 from *atg16l1^-/-^ ATG16L1 WT* and *atg16l1^-/-^ ATG16L1^T300A^* after stimulation with TNFα and CHX. (*n* = 5/4). GAPDH served as the loading control. (**G**) Quantification of CXCL1 in the supernatant of *atg16l1^-/-^*, *atg16l1^-/-^ ATG16L1 WT* and *atg16l1^-/-^ ATG16L1^T300A^* IECs after silencing with *siGpx4* and stimulation with the ω-6 PUFA AA for 24 h. (*n* = 4/8/4). Median is shown. Kruskal Wallis test with Dunn’s correction. **P<0.05.*


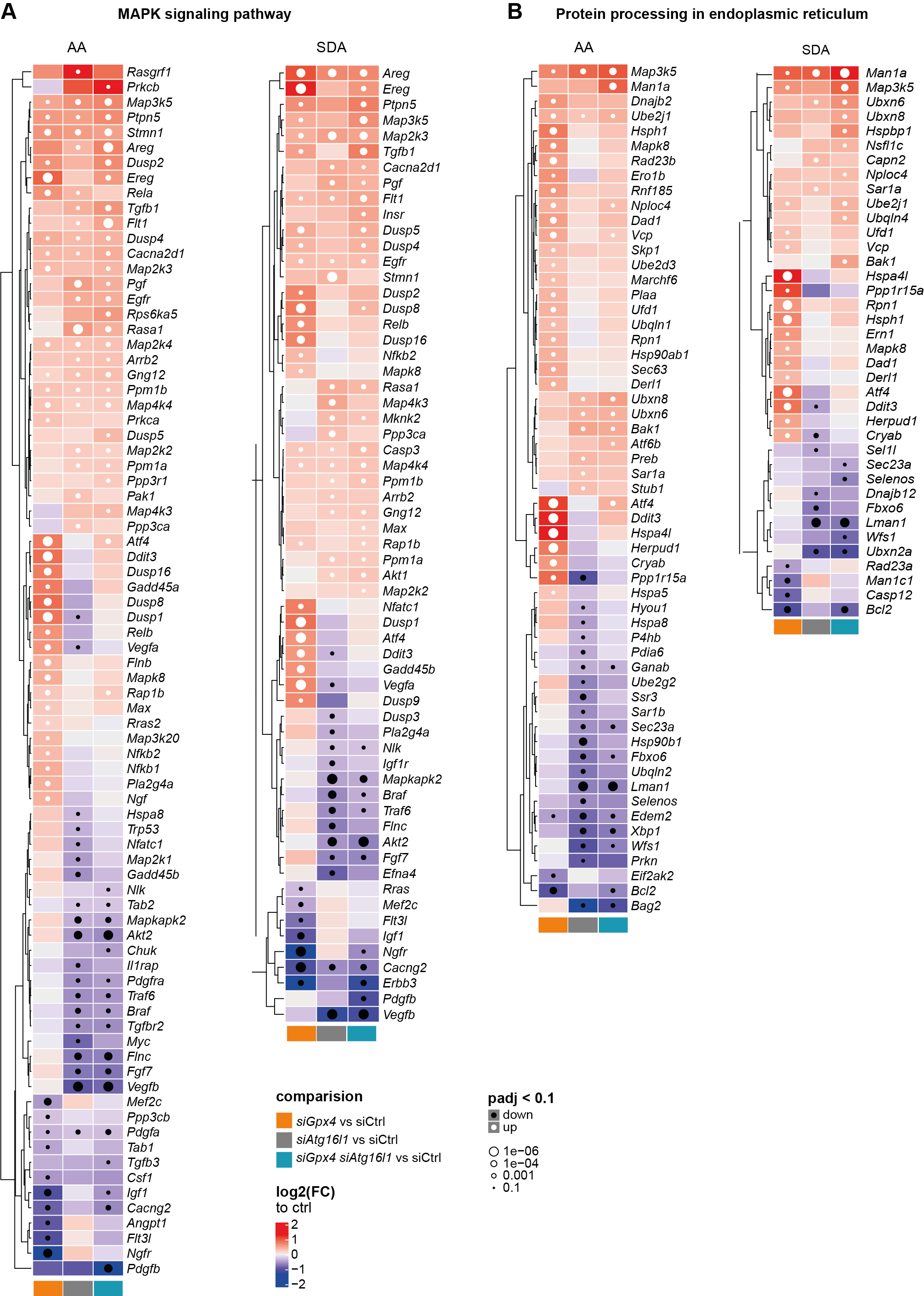


**Figure S5***. Atg16l1* is involved in MAPK signaling and protein processing at the endoplasmic reticulum during PUFA-induced CXCL1 production. (**A, B**) Heatmap showing significantly altered expression of MAPK signaling pathway genes (**A**) and genes involved in the protein processing at the endoplasmic reticulum (**B**) in *siGpx4* (orange), *siAtg16l1* (gray) and *siGpx4 siAtg16l1* (blue) after ω−6 PUFA (AA) and ω−3 PUFA (SDA) compared to siCtrl IECs. Genes are indicated on the right (*n* = 4).


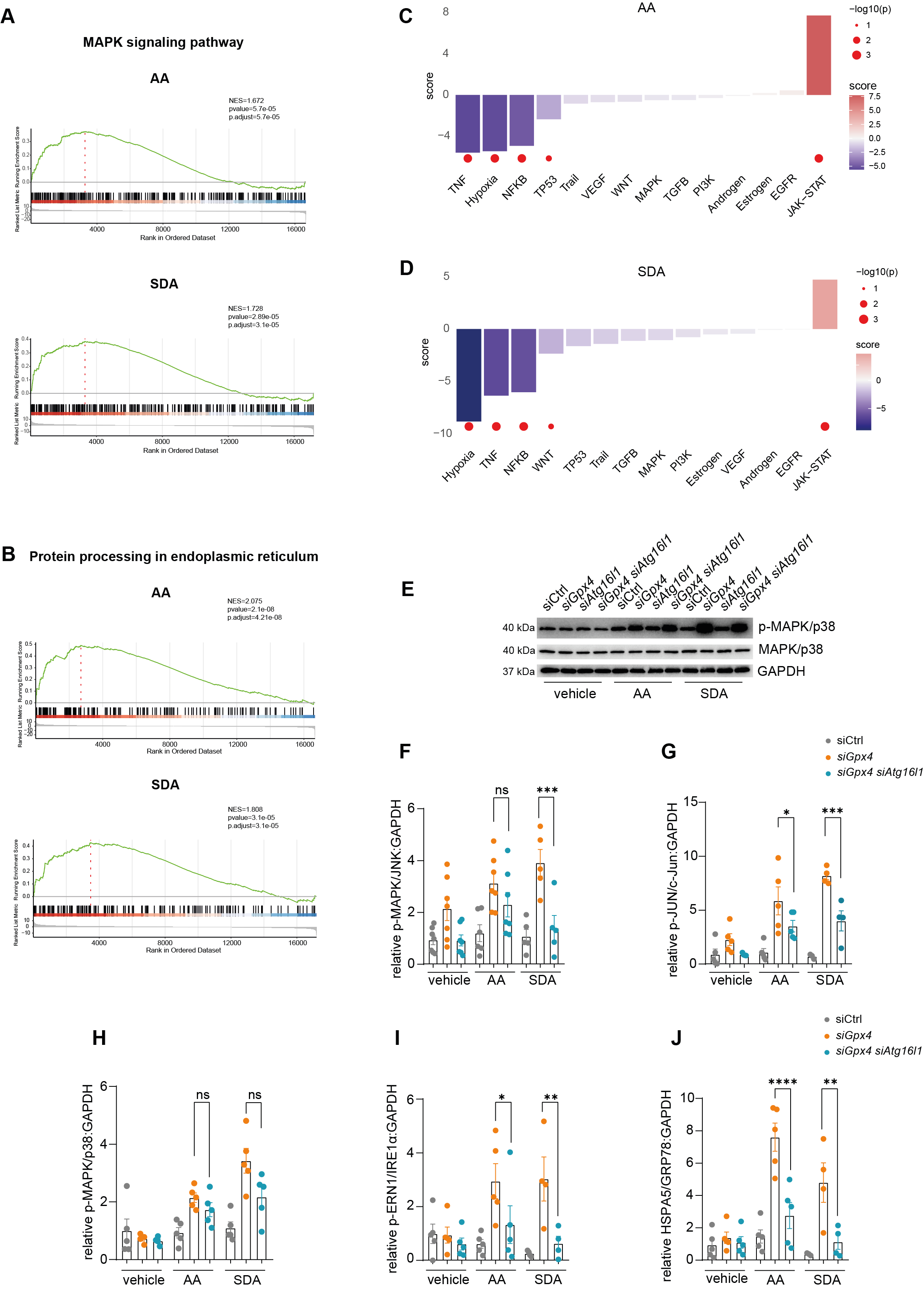


**Figure S6.** *Atg16l1* is required for PUFA-induced MAPK signaling and ER stress in *siGpx4* IECs. (**A, B**) Enrichment plots showing significant upregulation of MAPK signaling pathway (**A**) and the protein processing at the endoplasmic reticulum pathway (**B**) in AA and SDA stimulated IECs. *siGpx4* IECs were compared to siCtrl IECs (*n* = 4). Red and blue color bar: This bar corresponds to the ranked list of genes. * Red: Indicates genes in the MAPK signaling/ protein processing at the endoplasmic reticulum pathway that are upregulated in *siGpx4 siAtg16l1* +AA/+SDA compared to *siGpx4* +AA/+SDA. Blue: Indicates genes in the MAPK signaling/ protein processing at the endoplasmic reticulum signaling pathway that are downregulated in *siGpx4 siAtg16l1* +AA/+SDA compared to *siGpx4* +AA/+SDA. The intensity of the color reflects the degree of upregulation or downregulation. (**C, D**) barplots showing activity scores of 14 analyzed pathways in *siGpx4 siAtg16l1* IECs compared to *siGpx4* IECs after ω-6 PUFA (AA, **C**) or ω-3 PUFA (SDA, **D**) stimulation for 8 h. (*n* = 4). Pathways are indicated on the X-axis. (**E**) A representative immunoblot of (phospho-) MAPK/p38 in siCtrl, *siGpx4*, *siAtg16l1* and *siGpx4 siAtg16l1* IECs after ω-6 PUFA (AA) or ω-3 PUFA (SDA) stimulation for 24 h. GAPDH served as a loading control (*n* = 5). (**F - J**) Quantification of p-MAPK/JNK (**F**), p-JUN/c-Jun (**G**), p-MAPK/p38 (**H**), p-ERN1/IRE1α (**I**) and HSPA5/GRP78 (**J**) relative to GAPDH of siCtrl, *siGpx4* and *siGpx4* *Atg16l1* IECs determined by densitometry (*n* ≥ 4) of immunoblots shown in **Figure 6 C, E**. **P<0.05*, ***P<0.01,* ****P<0.001,* *****P<0.0001*.


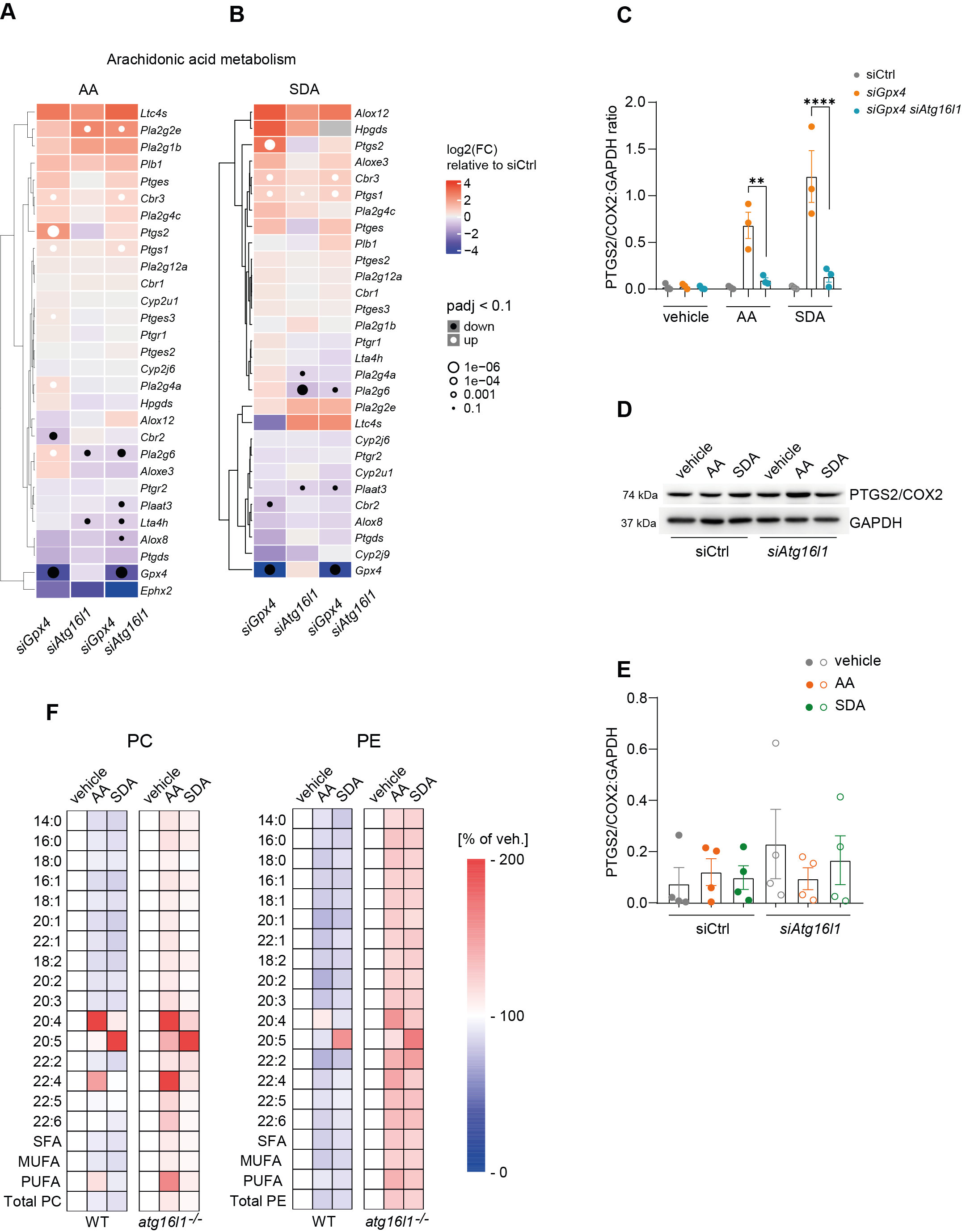


**Figure S7.** *Atg16l1* is involved in the regulation of PUFA metabolism in *siGpx4* IECs. (**A, B**) Heatmap showing altered expression of AA metabolism genes in *siGpx4*, *siAtg16l1* and *siGpx4* si*Atg16l1* IECs after stimulation with ω-6 PUFA (AA, **A**) or ω-3 PUFA (SDA, **B**) for 8 h compared to siCtrl. AA metabolism genes are indicated on the right. (**C**) Quantification of PTGS2/COX2 relative to GAPDH of siCtrl, *siGpx4* and *siGpx4* *Atg16l1* IECs determined by densitometry (*n* = 3) of immunoblot shown in **Figure 8D**. (**D, E**) A representative immunoblot (**D**) and quantification (**E**) of PTGS2/COX2 in siCtrl and *siAtg16l1* IECs after ω-6 PUFA (AA) or ω-3 PUFA (SDA) stimulation for 24 h. Representative of (*n* = 3) independent experiments. GAPDH served as the loading control. (**F**) Incorporation of AA, SDA or their elongation/desaturation products in PC and PE. The heatmap shows the fold change in the absolute amount of the indicated fatty acid subclasses (SFA, MUFA, PUFA) in PC (left) and PE (right) compared to vehicle control. Fold changes in total PC and PE were calculated by summarizing all respective phospholipids species analyzed. ***P<0.01,* *****P<0.0001*.


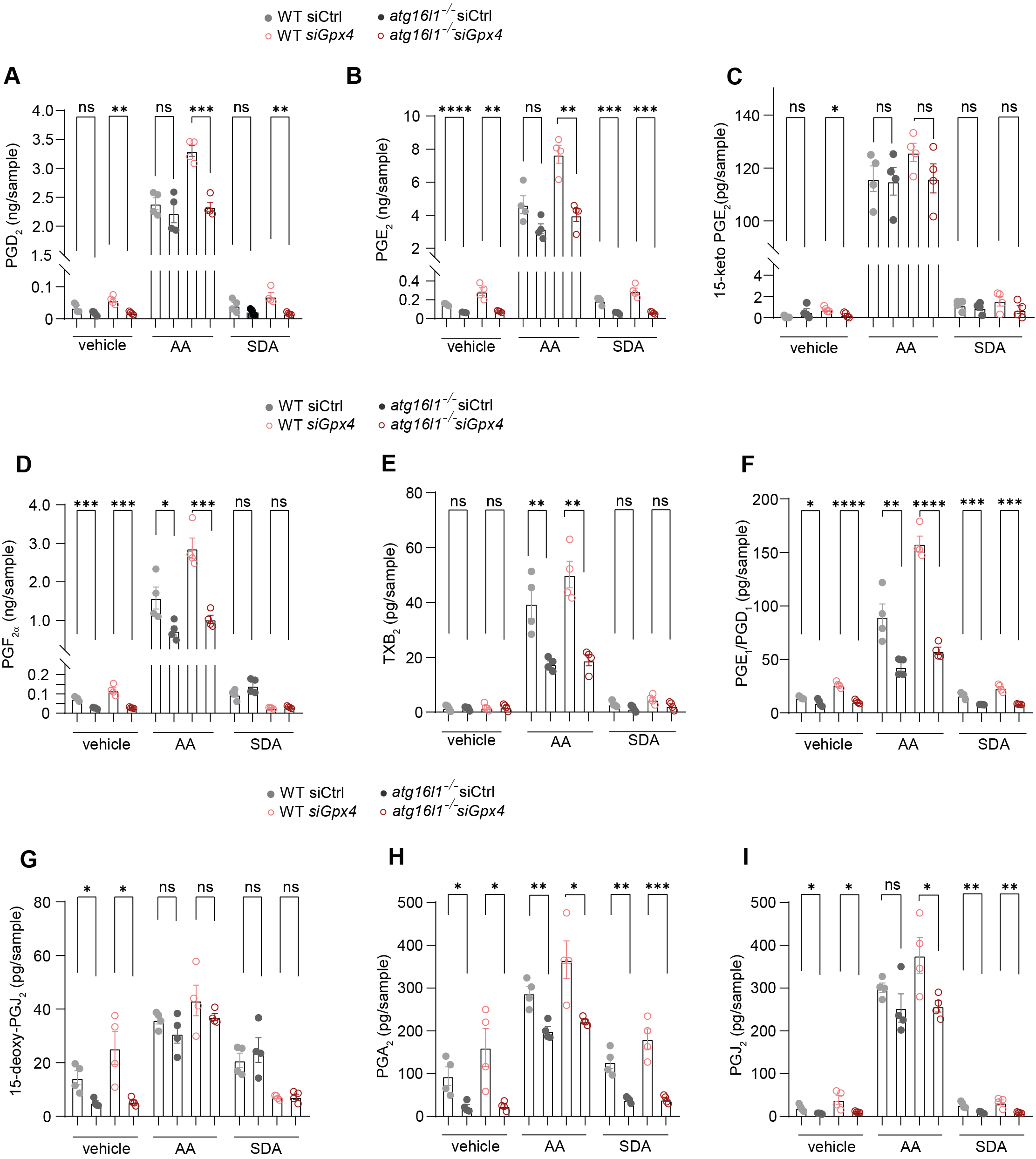


**Figure S8.** *atg16l1^-/-^* IECs are impaired in their ability to generate prostanoids. (**A - I**) Abundance of indicated lipid mediators produced by indicated IECs after stimulation with ω-6 PUFA (AA) or ω-3 PUFA (SDA) stimulation for 30 min as analyzed by UPLC-MS/MS (*n* = 4).

**P<0.05*, ***P<0.01,* ****P<0.001,* *****P<0.0001.*

*
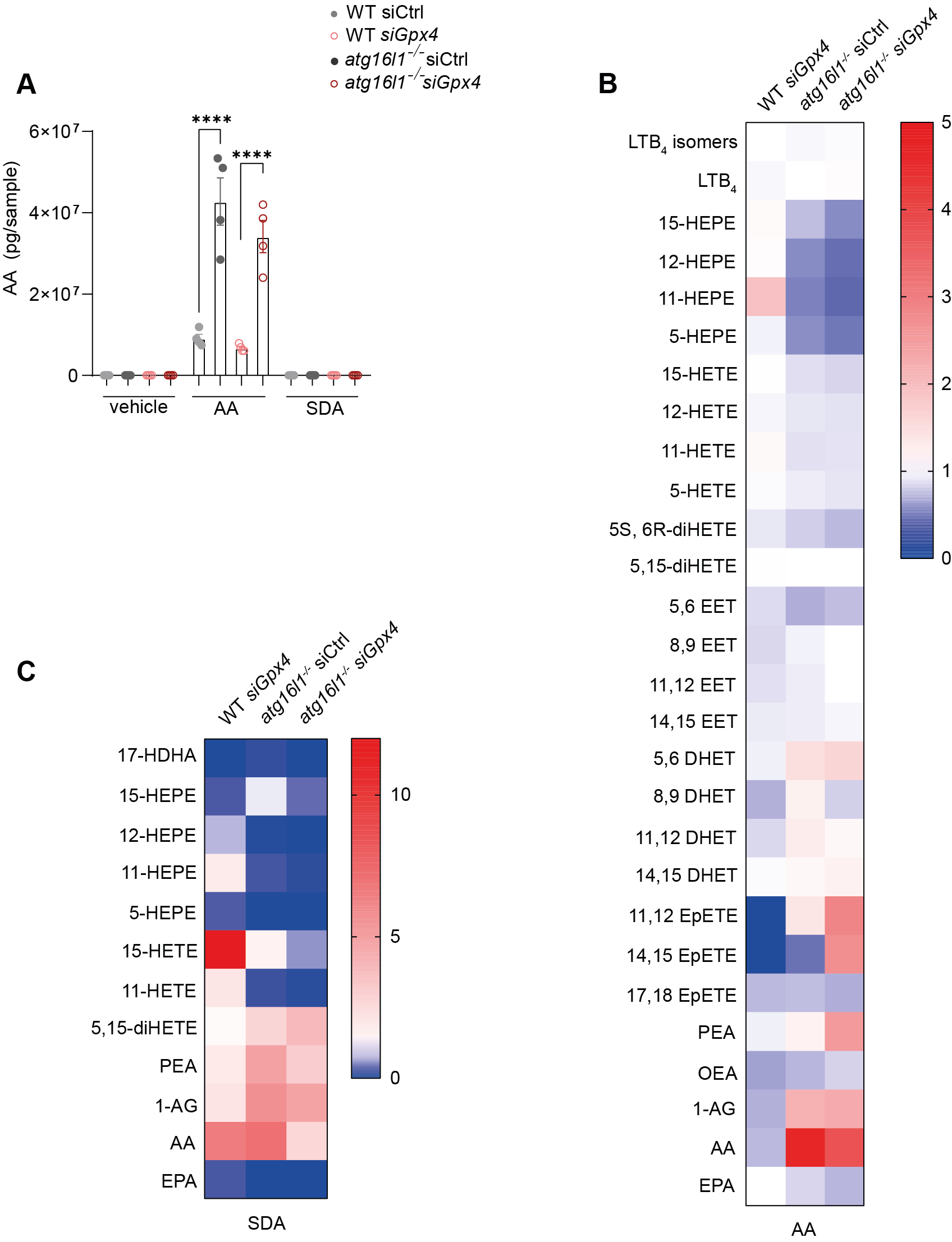
*

**Figure S9.** Effects of *Atg16l1*-deficiency on the lipid profile in IECs. (**A**) Quantification of AA in the supernatant of siCtrl and *siGpx4* WT and *atg16l1^-/-^* IECs after stimulation with ω-6 PUFA (AA) or ω-3 PUFA (SDA) stimulation for 30 min as analyzed by UPLC-MS/MS (*n* = 4). (**B, C**) Heatmaps showing fold-changes of lipid mediator levels of *siGpx4* WT and *atg16l1^-/-^* IECs after stimulation with ω-6 PUFA (AA) or ω-3 PUFA (SDA) for 30 min relative to WT siCtrl IECs, as analyzed by UPLC-MS/MS (*n* = 4).

*****P<0.0001.*
